# Teixobactin kills bacteria by a two-pronged attack on the cell envelope

**DOI:** 10.1038/s41586-022-05019-y

**Published:** 2022-08-03

**Authors:** Rhythm Shukla, Francesca Lavore, Sourav Maity, Maik G. N. Derks, Chelsea R. Jones, Bram J. A. Vermeulen, Adéla Melcrová, Michael A. Morris, Lea Marie Becker, Xiaoqi Wang, Raj Kumar, João Medeiros-Silva, Roy A. M. van Beekveld, Alexandre M. J. J. Bonvin, Joseph H. Lorent, Moreno Lelli, James S. Nowick, Harold D. MacGillavry, Aaron J. Peoples, Amy L. Spoering, Losee L. Ling, Dallas E. Hughes, Wouter H. Roos, Eefjan Breukink, Kim Lewis, Markus Weingarth

**Affiliations:** 1 NMR Spectroscopy, Bijvoet Centre for Biomolecular Research, Department of Chemistry, Faculty of Science, Utrecht University, Utrecht, The Netherlands; 2 Membrane Biochemistry and Biophysics, Bijvoet Centre for Biomolecular Research, Department of Chemistry, Faculty of Science, Utrecht University, Utrecht, The Netherlands; 3 Moleculaire Biofysica, Zernike Instituut, Rijksuniversiteit Groningen, Groningen, The Netherlands; 4 Department of Chemistry, University of California Irvine, Irvine, CA USA; 5 Magnetic Resonance Center (CERM) and Department of Chemistry ‘Ugo Schiff’, University of Florence, Sesto Fiorentino (FI), Italy; 6 Consorzio Interuniversitario Risonanze Magnetiche MetalloProteine (CIRMMP), Sesto Fiorentino, Florence, Italy; 7 Cell Biology, Neurobiology and Biophysics, Department of Biology, Faculty of Science, Utrecht University, Utrecht, The Netherlands; 8 NovoBiotic Pharmaceuticals, Cambridge, MA USA; 9 Antimicrobial Discovery Center, Department of Biology, Northeastern University, Boston, MA USA

**Keywords:** Mechanism of action, Drug development, Solid-state NMR, Antibiotics, Atomic force microscopy

## Abstract

Antibiotics that use novel mechanisms are needed to combat antimicrobial resistance^1–3^. Teixobactin^4^ represents a new class of antibiotics with a unique chemical scaffold and lack of detectable resistance. Teixobactin targets lipid II, a precursor of peptidoglycan^5^. Here we unravel the mechanism of teixobactin at the atomic level using a combination of solid-state NMR, microscopy, in vivo assays and molecular dynamics simulations. The unique enduracididine C-terminal headgroup of teixobactin specifically binds to the pyrophosphate-sugar moiety of lipid II, whereas the N terminus coordinates the pyrophosphate of another lipid II molecule. This configuration favours the formation of a β-sheet of teixobactins bound to the target, creating a supramolecular fibrillar structure. Specific binding to the conserved pyrophosphate-sugar moiety accounts for the lack of resistance to teixobactin^4^. The supramolecular structure compromises membrane integrity. Atomic force microscopy and molecular dynamics simulations show that the supramolecular structure displaces phospholipids, thinning the membrane. The long hydrophobic tails of lipid II concentrated within the supramolecular structure apparently contribute to membrane disruption. Teixobactin hijacks lipid II to help destroy the membrane. Known membrane-acting antibiotics also damage human cells, producing undesirable side effects. Teixobactin damages only membranes that contain lipid II, which is absent in eukaryotes, elegantly resolving the toxicity problem. The two-pronged action against cell wall synthesis and cytoplasmic membrane produces a highly effective compound targeting the bacterial cell envelope. Structural knowledge of the mechanism of teixobactin will enable the rational design of improved drug candidates.

## Abstract (web summary)

Using a combination of methods, the mechanism of the antibiotic teixobactin is revealed.

## Main

A rapid rise in multidrug-resistant bacteria is a major concern for global health^6,7^. This threat is exacerbated by a drought in the antibiotic pipeline^3,8^, with alarmingly few new classes of antibiotics introduced into the clinic over the past three decades.

**Fig. 1:**
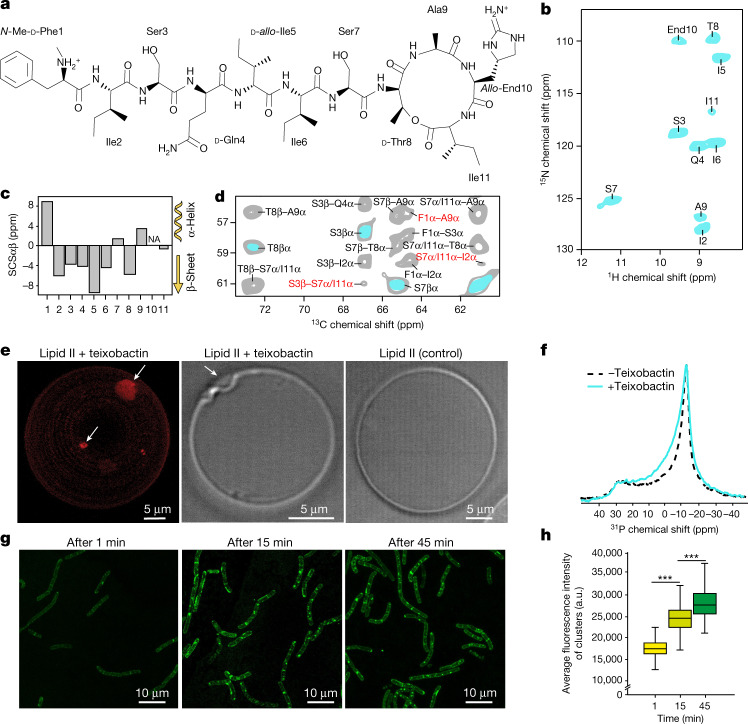
Oligomerization and membrane remodelling. **a**, Chemical structure of teixobactin. **b**, 2D NH ssNMR spectrum of lipid II-bound teixobactin in membranes. **c**, Secondary chemical shifts (SCS^23^) show β-structuring of the N terminus. Source data are provided. NA, not applicable. **d**, 2D CC ssNMR spectra of lipid II-bound teixobactin acquired with 50 ms (cyan) and 600 ms (grey) magnetization transfer time show intermolecular Cα–Cα contacts (red) consistent with the formation of antiparallel teixobactin β-sheets. **e**, Visualization of lipid II clustering (arrows) in 3D (left), using confocal microscopy of GUVs doped with Atto-labelled lipid II and treated with teixobactin. The transmission image of the same GUV revealed membrane perturbations induced by the oligomers (middle). Control GUVs with Atto-labelled lipid II, without teixobactin (transmission) (right) are also shown. Note that as the plane used for the middle image is not exactly in the middle of the GUV, the diameter is smaller than for the entire GUV as seen in the left image. The experiment was performed in biological triplicates. **f**, Static ^31^P ssNMR spectra show membrane perturbation induced by teixobactin (cyan line) in contrast to the untreated lipid II-doped DOPC large unilamellar vesicles (black dashed line). **g**, Confocal microscopy with *B. megaterium* incubated in the presence of a fluorescent teixobactin analogue^26^. Intense, elongated teixobactin clusters form fast and get more compact after 45 min. **h**, Boxplot of the fluorescence intensity of clusters after 1, 15 and 45 min of incubation with teixobactin. *n* = 380 clusters from biological triplicates. Data are represented as a boxplot in which the middle line is the median, the lower and upper hinges correspond to the first and third quartiles, the upper whisker extends from the hinge to the largest value and the lower whisker extends from the hinge to the smallest value, no further than 1.5× interquartile range. ***P < 0.0001, unpaired, two-tailed Student's *t*-test. Source data

In 2015, screening of uncultured bacteria from soil samples unearthed teixobactin^4^, a novel antibiotic with broad activity against multidrug-resistant Gram-positive pathogens such as methicillin-resistant *Staphylococcus aureus*, *Streptococcus pneumoniae* and vancomycin-resistant Enterococci^4^. Studies in animal models of infection suggest that teixobactin is a promising drug lead^4,9,10^. Teixobactin is an undecapeptide that contains five non-canonical amino acids, including four d-amino acids and the cationic l-*allo*-enduracididine (End10) localized in a C-terminal depsi-cycle (Fig. 1a and Supplementary Fig. 1). Enduracididine contains a unique five-membered cyclic guanidinium moiety that is rarely found in nature^11^ and its prominent position at the putative lipid II-binding interface has sparked considerable interest. Several groups have accomplished the complicated synthesis of teixobactin^12–14^ or its analogues^15–17^; however, they have not resolved the role of End10 in the mode of action.

Recently, we presented a structural model of the fibril-like complex formed between the synthetic analogue R4L10-teixobactin and lipid II using solid-state NMR (ssNMR)^18^, which enables the study of membrane-acting drugs under native conditions^19^. However, no structural information could be obtained for the non-canonical amino acids as these residues were either replaced or could not be ^13^C,^15^N-labelled in the synthetic teixobactin and were thus inaccessible by ssNMR. Thus, it remains unclear what role l-*allo*-enduracididine and other residues such as the cationic *N*-methyl-d-phenylalanine (Phe1)^20,21^ have in binding to the target. We also showed that R4L10-teixobactin–lipid II oligomerized into clusters on membrane surfaces^18^. However, the role of oligomerization in the action of teixobactin remains unclear.

## Oligomerization and membrane damage

**Extended Data Fig. 1:**
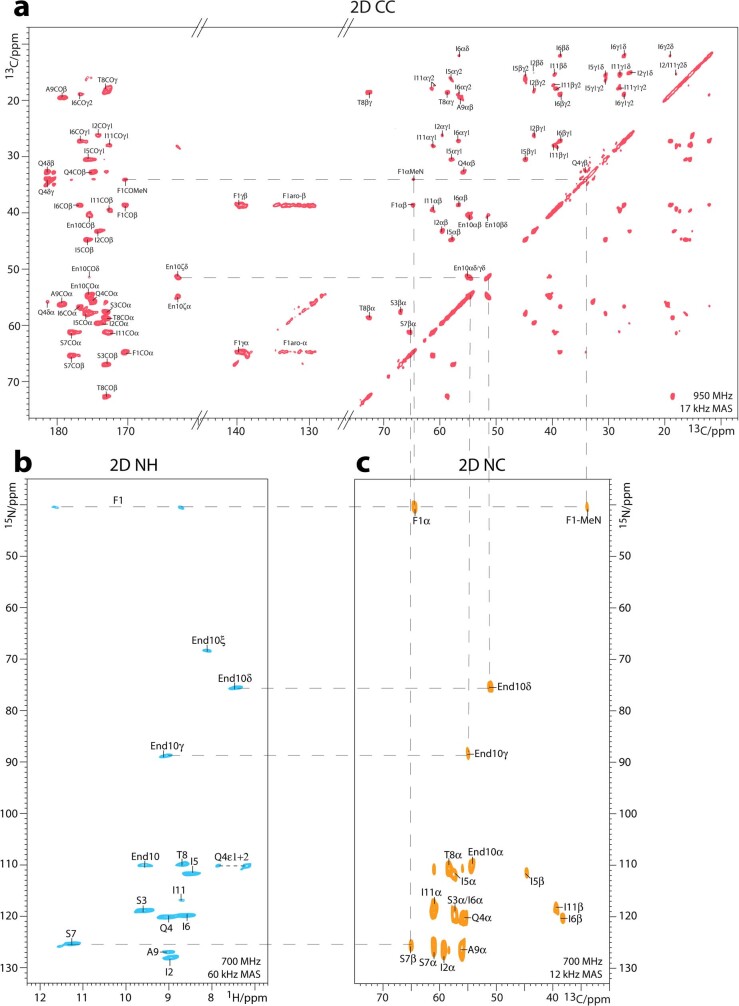
High-resolution ssNMR studies of the teixobactin - Lipid II complex in membranes. All ssNMR spectra were acquired with uniformly ^13^C,^15^N-labelled teixobactin bound to ^12^C,^14^N-Lipid II in DOPC liposomes. a) 2D ^13^C^13^C PARISxy^33^ spectrum acquired at 950 MHz and 18 kHz MAS using 50 ms CC magnetization transfer time. All ^13^C nuclei of teixobactin are assigned. b) 2D NH spectrum acquired at 700 MHz and 60 kHz MAS. All signals are assigned. c) 2D CN spectrum acquired at 700 MHz and 12 kHz MAS using 5 ms N to C cross-polarization time. Some connectivities, including for the End10 sidechain, are shown by dashed lines. Note that our 2D NH data of the ^13^C,^15^N-teixobactin – Lipid II complex in membranes match well to a previously published spectrum of the complex in dodecylphosphocholine (DPC) micelles^22^. Interestingly, compared to micellar assignments, we observe major (>5 ^13^C ppm) local differences for the two residues that most prominently interact with Lipid II, something that could suggest an impact of the membrane environment for complex formation. See Supplementary Table 3 for a comparison of chemical shifts of the teixobactin – Lipid II complex in micelles and in membranes. Note that backbone assignments were conclusively validated with a pair of ^1^H-detected 3D CαNH/CONH experiments^65^.

**Extended Data Fig. 2:**
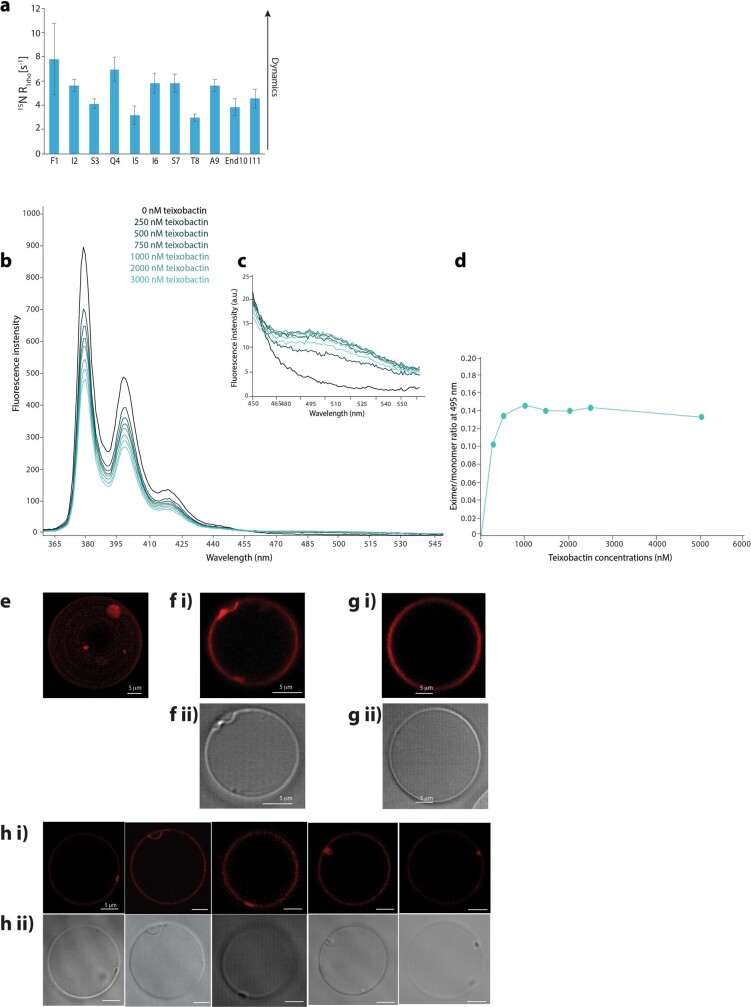
Dynamics of teixobactin in the complex and pyrene excimer fluorescence confirm Lipid II oligomerization upon binding of teixobactin. a) Site-resolved ^15^N T_1rho_ dynamics of Lipid II-bound teixobactin in membranes acquired at 60 kHz (MAS). The overall dynamics of the molecule is very slow, in agreement with the immobilization of teixobactin in large oligomeric complexes. The error bars indicate the standard error of the fit. Data represented as mean +/− SD. Source data are provided. Monomeric pyrene fluorescence emission maxima occur at 378, 398, and 417 nm. Pyrene molecules exhibit a unique fluorescence at 490 nm only when two pyrene molecules come within 10-20 Å of each other, forming an excited dimer state known as the excimer state. Source data are provided. b) Fluorescence spectra of pyrene-labelled Lipid II in LUVs for increasing concentrations of teixobactin. It can be readily seen that with the increase in teixobactin concentration there is a relative decrease in the monomer signal followed by a respective increase in the excimer signal. Each spectrum is acquired immediately after the addition of teixobactin and averaged over 50 s, demonstrating that the oligomerization event is almost instantaneous. c) The insert shows the excimer region of the plot. d) Quantification of the excimer over monomer ratio as a function of the teixobactin concentration. e) Fluorescence microscopy shows oligomerization and membrane perturbations. A 3D render of the z-stack of DOPC GUVs doped with Atto-labelled Lipid II and treated with teixobactin shows Lipid II oligomerization. f) A 2D plane of the same GUV shown in e) revealed membrane perturbations induced by the oligomers. ii) Transmission image of the GUV. g) Control GUV: i) fluorescent ii) Transmission image; h) i) Confocal microscopy images of GUVs taken from biological triplicates ii) The transmission images. All scale bars represent 5 μm. Source data

**Extended Data Fig. 3:**
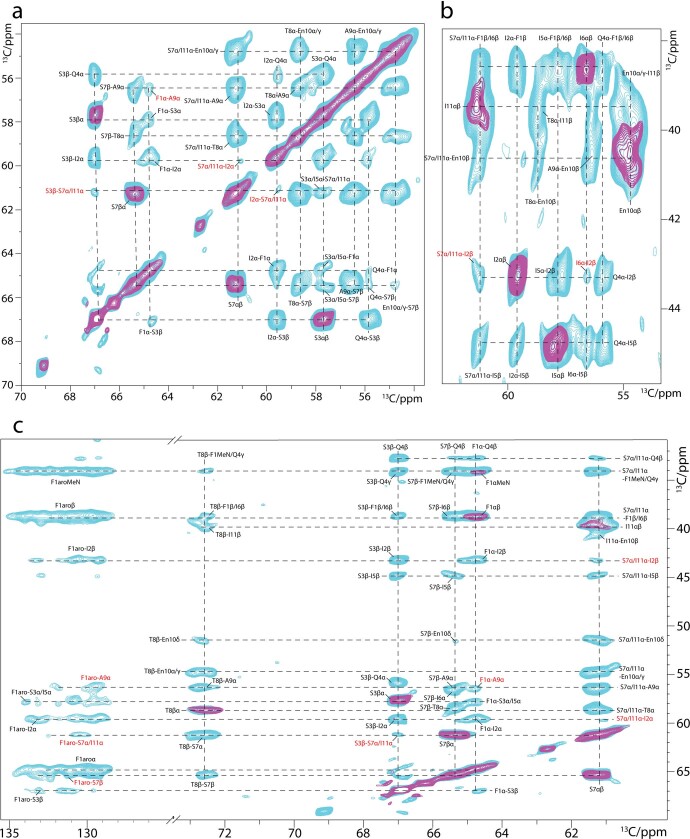
Intermolecular teixobactin – teixobactin interactions seen by ssNMR. Solid-state NMR ^13^C-^13^C PARISxy spectra of the ^13^C,^15^N-teixobactin – Lipid II complex in membranes show several cross-peaks (in red) that cannot be intramolecular contacts because this would require magnetization transfer over more than 10 Å. Rather, these cross-peaks correspond to intermolecular contacts between different teixobactin molecules and agree with the formation of antiparallel β-sheets. Spectra were acquired at 950 MHz and 18 kHz MAS using 50 (purple) and 600 (cyan) ms CC magnetization transfer. a) and b) show zooms into spectral Cα-Cα and Cα-Cβ regions, while c) shows long-range interactions with the aromatic sidechain of Phe1.

**Extended Data Fig. 4:**
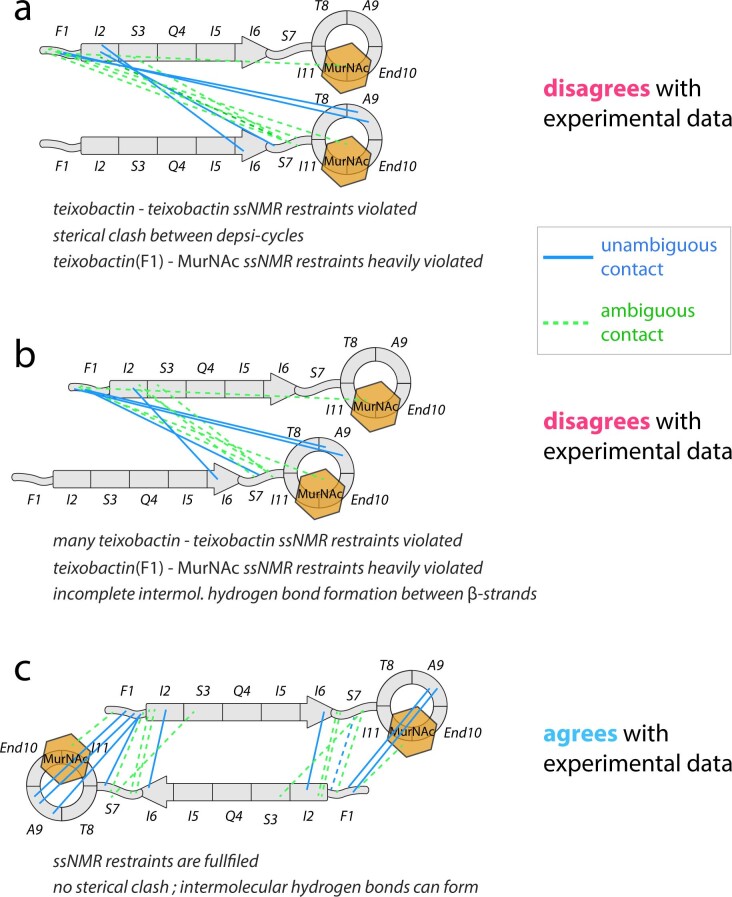
Only an antiparallel arrangement agrees with ssNMR data. ssNMR data (Fig. 1d and Extended Data Fig. 3) provide clear evidence that teixobactin molecules oligomerize upon Lipid II binding and form β-sheets. We confirm oligomerization by several microscopy setups *in vitro* and *in vivo*. ssNMR data support that teixobactin molecules feature antiparallel arrangement in oligomers. Unambiguous NMR contacts are shown as continuous blue lines; ambiguous contacts are shown as dashed green lines. For ambiguous contacts, only the contact with the shorter average distance is shown (see Supplementary Tables 5–7). a) *Parallel* arrangement. This can be ruled out because most intermolecular teixobactin – teixobactin ssNMR contacts would be >>10 Å and the interfacial contact between F1 of teixobactin and MurNAc of Lipid II would be >>20 Å, while the maximum threshold for ssNMR ^13^C-^13^C distance measurements is ~8 Å. Moreover, the depsi-cycles of adjacent teixobactin molecules would clash. b) *Shifted parallel* arrangement. Steric clashes are avoided, but intermolecular contacts between teixobactin molecules are heavily violated, and contacts between the N-terminus of teixobactin (F1) and Lipid II (MurNAc) remain heavily violated. In addition, the formation of teixobactin – teixobactin hydrogen bonds is compromised. c) *Antiparallel arrangement*. All Intermolecular and interfacial ssNMR restraints are fulfilled. There are no steric clashes and intermolecular hydrogen bonds can form. An antiparallel arrangement also agrees with the X-ray structure of a teixobactin-sulfate complex in solution^66^. Contacts consistent with antiparallel β-sheets are listed in Supplementary Table T4. Note that some contacts are ambiguous: Contacts with S7α could also be with I11Cα. Note that contacts with I11α would also (even more than with S7α) only be feasible in antiparallel sheets. Likewise, interfacial contacts between MurNAc and F1MeN could also be with Q4γ. Here also, the distance Q4γ - MurNAc – would be >>10 Å in parallel β-sheets.

To make the entire antibiotic amenable to ssNMR studies, we produced uniformly ^13^C,^15^N-labelled^22^ natural teixobactin in its native host *Eleftheria terrae*. Teixobactin and lipid II form a well-defined complex in liposomes that yields high-quality ssNMR spectra (Fig. 1b and Extended Data Fig. 1a–c). An analysis of the chemical shifts^23^ shows that the N terminus (Ile2–Ile6) adopts β-strand conformation in the bound state (Fig. 1c), in line with its high rigidity (Extended Data Fig. 2a). We previously reported on a synthetic teixobactin analogue in which β-structuring is caused by the formation of antiparallel teixobactin β-sheets^18^. For natural teixobactin, a 2D ssNMR ^13^C–^13^C spectrum with a long magnetization transfer time that probes distances with a threshold of approximately 8–9 Å (Fig. 1d and Extended Data Fig. 3) strongly supports that natural teixobactin also forms antiparallel β-sheets. We observed an unambiguous head-to-tail contact between Phe1Cα and Ala9Cα. In addition, we observed contacts between N-terminal (Phe1, Ile2 and Ser3) and C-terminal (Ser7 or Ile11) residues. Although we could not resolve whether these contacts were with Ser7 or Ile11, all contacts must be intermolecular given that the distance between these residues within the same β-strand is 12–18 Å and hence well above the NMR distance threshold, and all of these contacts are consistent with antiparallel β-sheets, but not with parallel β-sheets (Extended Data Fig. 4). Using pyrene-tagged lipid II^24^, we confirmed that oligomers form immediately after the addition of teixobactin (Extended Data Fig. 2b,c). Our data hence demonstrate that oligomerization upon target binding is necessary for the high potency of teixobactin, as N-terminally truncated constructs have drastically reduced activity^21^.

We used confocal microscopy to probe the accumulation of the complex on the surface of giant unilamellar vesicles (GUVs) doped with Atto 550-tagged lipid II (Fig. 1e and Extended Data Fig. 2e–h). Microscopy images clearly show the formation of micron-sized teixobactin–lipid II oligomers. Remarkably, in 2D slices from the z-stack of the GUVs, we observed pronounced concave membrane perturbations at the oligomerization sites, whereas the rest of the GUV surface maintained a regular morphology. We performed static ^31^P ssNMR spectroscopy to confirm that lipid II-induced oligomerization causes membrane defects^25^ (Fig. 1f). Although we obtained the characteristic signal pattern of lamellar membranes in the absence of the drug, incubation with teixobactin markedly changed the signal.

To examine whether teixobactin oligomerization also occurs in bacteria, we visualized *Bacillus megaterium* cells with a fluorescent teixobactin analogue^26^ using confocal microscopy (Fig. 1g,h). The formation of clusters in bacteria was fast and pronounced; elongated supramolecular teixobactin structures were observed within 15 min. After 45 min of incubation, clusters showed a more compact shape. However, the small increase in the fluorescence intensity implies that most teixobactin molecules are already involved in clusters after 15 min.

**Fig. 2:**
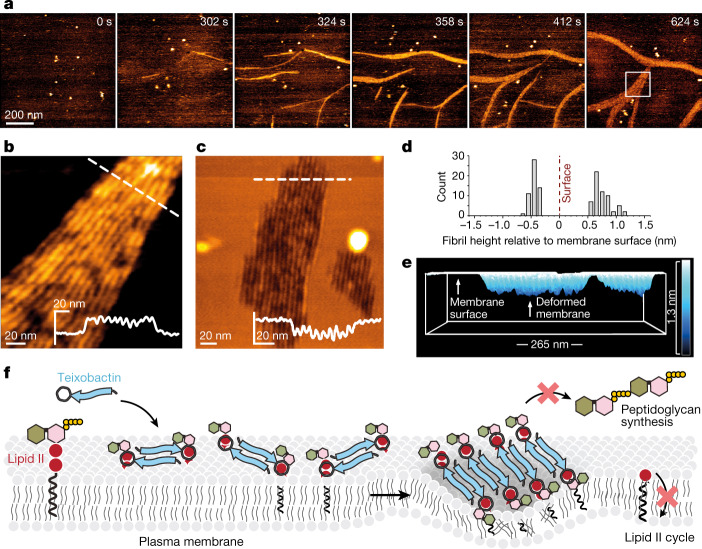
Teixobactin sequesters lipid II into supramolecular fibrils. **a**, Snapshots of a timelapse HS-AFM video (Supplementary Video 1) following the assembly of teixobactin–lipid II fibrils. Images were obtained on a supported lipid bilayer containing 1% (mol) lipid II in the presence of 800 nM teixobactin, added after 24 s. Image acquisition rate of 0.5 frames per second. **b**, Zoomed in view of an HS-AFM image of the fibrillar sheet on the membrane surface, as marked by a white rectangle in **a** at 624 s. The inset in the lower right corner shows the height profile at the dashed line. **c**, HS-AFM image of a lipid bilayer deformed by teixobactin–lipid II fibrils below the membrane surface, 50 min after the addition of 800 nM teixobactin. The inset shows the height profile at the dashed line. **d**, Histogram showing the relative height of teixobactin–lipid II fibrils above (measured directly when fibrils assemble on the membrane surface) and below (measured 50 min after the addition of 800 nM teixobactin) the lipid bilayer surface (*n* > 50). **e**, Side view of a 3D-rendered image in **c**. **f**, Model of the mode of action of teixobactin. Teixobactin first forms small β-sheets upon binding of lipid II, then elongates into fibrils that eventually associate into lateral fibrillar sheets, obstructing biosynthesis of peptidoglycan and causing membrane defects. Source data

**Extended Data Fig. 5:**
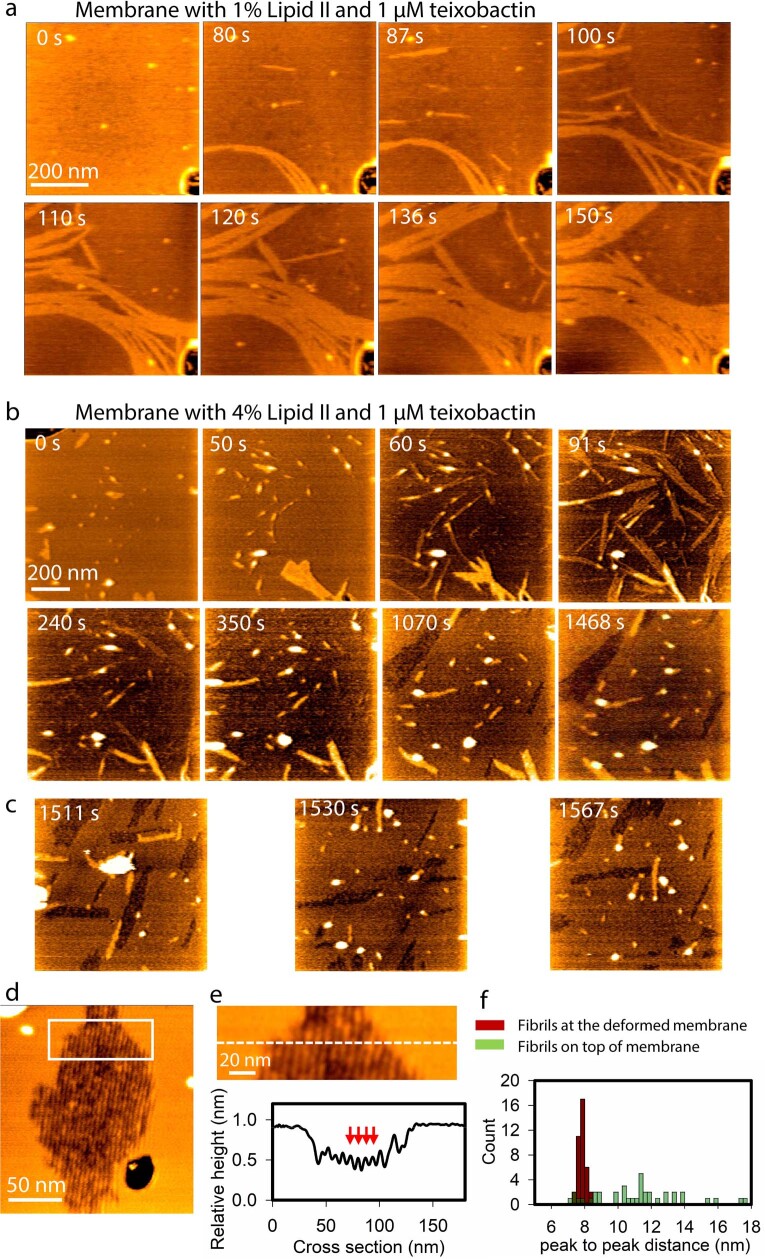
Growth of teixobactin-Lipid II fibrils and membrane deformation captured by HS-AFM. a) Snapshots of time-lapse High-Speed Atomic Force Microscopy (HS-AFM) images following the assembly of teixobactin-Lipid II fibrils on a supported DOPC-lipid bilayer in the presence of 1% Lipid II and 1 µM teixobactin. Image acquisition rate 2 frames/second. b) Snapshots of time-lapse HS-AFM images following the assembly of fibrils on a supported lipid bilayer in the presence of 4% Lipid II and 1 µM teixobactin. Fibril growth on the membrane (0-91 s), absorption of the fibrils (91-350 s), and membrane deformation (1070-1468 s) can be observed. In the last two frames both fibrillar sheets on top (yellow colour) and below (dark brown/black colour) the membrane can be observed. Image acquisition rate 2 frames/second. c) Snapshot images from the neighbouring regions (of the area panel b) that were not previously imaged. This shows that membrane deformation occurred over the entire surface independent of the imaging. d) Fibrillar sheet below the membrane, observed 50 min after the addition of 800 nM teixobactin on a membrane containing 1% Lipid II. The dark brown structures spanning from the top of the image to the bottom are the fibrils below the membrane (yellow color). The black area at the bottom right is a hole in the membrane where the mica surface can be observed. e) Zoom-in of white rectangle in panel d) including height profile of the fibrillar sheet below the membrane. The red arrows point at the tops of a few adjacent fibrils. f) Histogram of peak-to-peak distance between fibrils in sheets on top of the membrane and below the membrane surface revealing a stable ~8 nm peak-to-peak distance after the fibrils have descended into the surface. Source data

**Extended Data Fig. 6:**
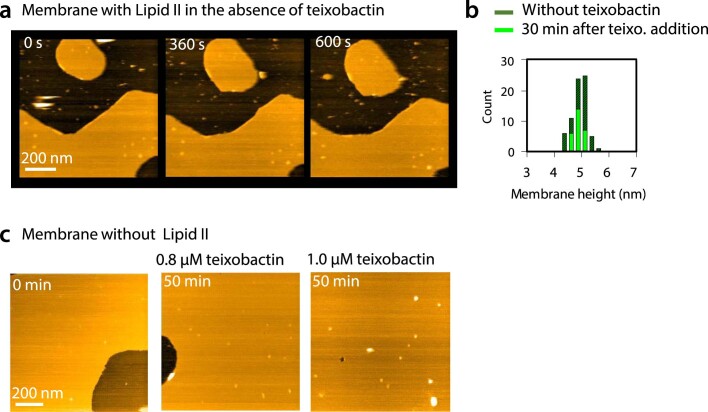
HS-AFM control experiments. Control experiments show that fibrils solely form in the presence of both teixobactin and Lipid II, and that membrane height at positions without fibrils remains unaltered. a) Snapshots of HS-AFM images of supported lipid bilayer with Lipid II (1 %) in the absence of teixobactin. Image acquisition rate 0.5 frames/second. b) Histogram representing membrane heights before and after (30 min) the addition of teixobactin. c) HS-AFM images of a DOPC-membrane without Lipid II in the absence of teixobactin (0 min) and 50 min after the addition of different concentrations of teixobactin. Source data

Next, we used high-speed atomic force microscopy (HS-AFM) to study the formation of teixobactin–lipid II oligomers in real time^27–29^. Within minutes after the addition of teixobactin to lipid membranes doped with lipid II, HS-AFM data show the formation of fibrils on the membrane surface, with a height of 0.8 ± 0.1 nm (Fig. 2a,b, Extended Data Figs. 5 and 6 and Supplementary Videos 1 and 2). Fibrils then associated or laterally folded onto each other, forging a sheet of fibrils, in line with the more compact clusters that we observed at 45 min in bacteria by confocal microscopy (Fig. 1g). Fibrils were solely observed in the presence of both teixobactin and lipid II (Extended Data Figs. 5 and 6). Although fibrils initially formed on top of the membrane, they afterwards descended into the membrane. This caused a sizeable membrane thinning; the fibrillar sheets stabilized approximately 0.5 nm below the membrane surface (Fig. 2c–e and Supplementary Video 3).

Together, HS-AFM, confocal microscopy, fluorescence spectroscopy and ssNMR data demonstrate that teixobactin uses a unique, sequential binding mode that results in the formation of fibrillar complexes that perturb the membrane (Fig. 2f). Initially, in a fast step, teixobactin forms antiparallel β-sheets upon binding of lipid II. These small oligomers then elongate into long fibrils that laterally associate and form compact fibrillar sheets. This mechanism sequesters lipid II, making it unavailable to peptidoglycan biosynthesis. In addition, the formation of clusters favourably affects the pharmacodynamics. The supramolecular fibrils were stable for hours in microscopy experiments and presumably form irreversibly on biological timescales given that samples of the complex yielded similar ssNMR spectra after months of storage at 278 K. Hence, the residence time (inverse of the dissociation rate constant (1/*k*_off_)) of teixobactin at its target site is presumably long, enhancing its biological activity^30^. This also means that teixobactin occupies its target long after the administration of the drug, which could prolong its action and be beneficial for the treatment of slow-growing bacteria^31^.

## The complex interface

**Fig. 3:**
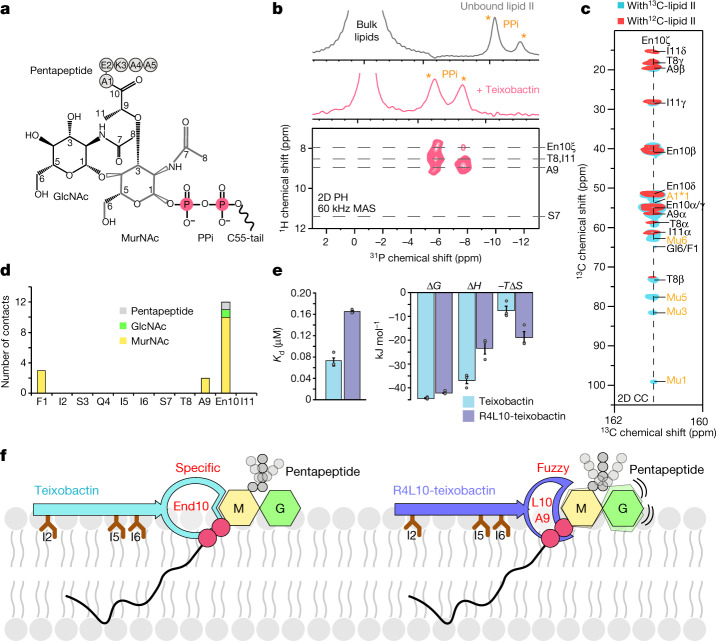
Enduracididine governs the interface. **a**, Chemical structure of lipid II. **b**, 1D ^31^P ssNMR data in liposomes show strong shifts of the lipid II PPi signals upon addition of teixobactin. 2D ^1^H^31^P (HP) ssNMR shows a direct interaction between the PPi group and the backbone amino-protons of the depsi-cycle and the sidechain of End10. MAS, magic angle spinning. **c**, Superposition of 2D ^13^C^13^C (CC) spectra of the complex with NMR-invisible ^12^C,^14^N-labelled (red) and NMR-active ^13^C,^15^N-lipid II (cyan) show a dominant presence of enduracididine at the interface. The cyan spectrum was acquired with a magnetization transfer time of 300 ms. Interfacial contacts with MurNAc and A1 of the pentapeptide are shown in orange. **d**, Sum of interfacial ssNMR contacts with the headgroup of lipid II. **e**, Binding energetics of the interface obtained by isothermal titration calorimetry. Data show the averages of three experiments for each drug. Data represented as mean ± s.e.m. Source data are provided as a Source Data file. **f**, Illustration of the differential binding modes of natural teixobactin (left) and the synthetic analogue R4L10-teixobactin^18^. Enduracididine in teixobactin specifically binds to MurNAc, which stabilizes the entire interface. The substitution of End10 by a leucine residue leads to a fuzzy interface with the headgroup of lipid II and reduces the binding affinity. Source data

We used ssNMR to determine the teixobactin–lipid II interface. Lipid II (Fig. 3a) is composed of a conserved sugar-pyrophosphate (GlcNAc–MurNAc–PPi) part and a pentapeptide whose variation confers resistance to glycopeptide antibiotics such as vancomycin^32^. We used magic angle spinning ^31^P ssNMR to examine the interaction between teixobactin and the PPi of lipid II (Fig. 3b). A 1D ^31^P ssNMR spectrum with lipid II-doped liposomes showed a stark signal shift of PPi upon addition of teixobactin. A 2D ^1^H^31^P ssNMR spectrum acquired with a short (1 ms) ^1^H to ^31^P magnetization transfer time demonstrated that the backbone amino protons of the depsi-cycle (Thr8-Ile11) directly coordinate the PPi group. A 2D ^1^H^31^P spectrum with a longer (2 ms) transfer time showed that the cationic End10 sidechain is in the direct vicinity of the anionic PPi, suggesting a favourable electrostatic interaction.

Next, we determined how teixobactin interacts with the sugar-pentapeptide headgroup of lipid II. For this, we assembled the complex with ^13^C,^15^N-lipid II^18^ and acquired 2D ^13^C^13^C PARISxy^33^ spectra (Fig. 3c). We obtained many interfacial contacts between teixobactin and the MurNAc sugar that is covalently attached to the lipid II PPi group, whereas we could not detect a single clear interfacial contact for the GlcNAc sugar (Fig. 3d). These data show that MurNAc is directly at the interface, whereas GlcNAc is distal. Hence, there is a global resemblance between the lipid II interfaces formed by teixobactin and by synthetic R4L10-teixobactin^18^, for none of which GlcNAc is a direct interaction partner. However, the interaction with MurNAc is strikingly different for natural teixobactin.

**Extended Data Fig. 7:**
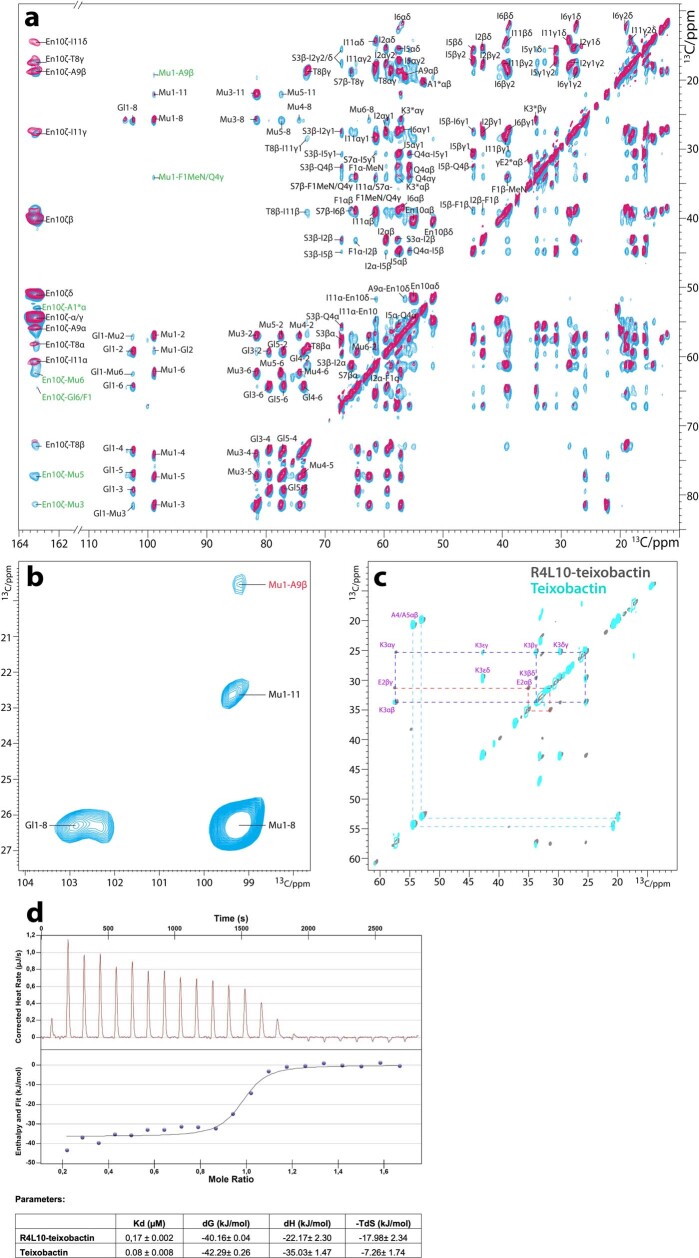
ssNMR analysis of the teixobactin – Lipid II interface and characterisation of the pentapeptide of Lipid II in the complex. a) 2D CC PARISxy^33^ spectra of ^13^C,^15^N-teixobactin and ^13^C,^15^N-Lipid II in DOPC liposomes acquired at 950 MHz using 150 (red) and 300 ms (blue) magnetization transfer. Interfacial contacts are labelled in green. Lipid II pentapeptide signals are marked with an asterisk, e.g., A1*αβ. b) Zoom into the spectrum acquired with 300 ms magnetization transfer. Only MurNAc shows a contact with Ala9 of teixobactin depsi-cycle, while GlcNAc is distal from the interface. c) Experiments that rely on scalar couplings can be used to detect highly mobile residues. The spectrum is based on a scalar 2D ^13^C^13^C TOBSY^35^ ssNMR experiment with the complex formed by ^13^C,^15^N-teixobactin and ^13^C,^15^N-Lipid II in liposomes acquired at 950 MHz, 8 kHz MAS, and 300 K temperature. Teixobactin and the Lipid II sugars are not visible in the spectrum (cyan). Conversely, the last three residues (Lys3-Ala4-Ala5) of the Lipid II pentapeptide give scalar signals indicating high mobility and no involvement in complex formation. While signals of E2 (red) of Lipid II are absent for the complex formed by natural teixobactin, these signals were previously^18^ observed on R4L10-teixobactin (grey). Likewise, the K3αγ signal of the third pentapeptide residue was only visible for R4L10-teixobactin. This is due to the tight binding of End10 of natural teixobactin to MurNAc, resulting in the rigidification of the first two residues of the pentapeptide. Conversely, pentapeptide residues A1 and E2 give well-defined dipolar signals for natural teixobactin. Residue K3 is visible in both dipolar and scalar spectra, implying intermediate mobility. d) A representative ITC thermogram showing a strong binding of natural teixobactin to Lipid II containing DOPC LUVs. All experiments were performed in triplicates. All the parameter values are shown as mean +/− SD.

Although the hydrophobic residues Ala9 and Leu10 of R4L10-teixobactin form a loosely defined fuzzy interface with MurNAc, natural teixobactin engages in tight and specific interactions due to the presence of End10, demonstrated by the 11 interfacial End10–MurNAc ssNMR contacts (Fig. 3d and Extended Data Fig. 7a,b). Some interfacial contacts were already visible with a short ^13^C^13^C magnetization transfer time of 150 ms, which is clear evidence of a tight interface. The tighter binding of natural teixobactin to lipid II could be confirmed by isothermal titration calorimetry (Fig. 3e and Extended Data Fig. 7d). Compared to R4L10-teixobactin (dissociation constant (*K*_d_) = 0.17 μM ± 0.002), natural teixobactin has a markedly higher binding affinity for lipid II (*K*_d_ = 0.08 μM ± 0.008) with a substantially more favourable binding enthalpy (Δ*H*) that most likely relates to additional hydrogen bonds and electrostatic interactions between End10 and lipid II. Moreover, we observed signals consistent with interfacial contacts between Phe1 and MurNAc in 2D ^13^C^13^C spectra, strongly suggesting that the cationic N terminus, which has been shown to be critical for killing activity^20,21^, is directly involved in the coordination of lipid II.

Teixobactin did not bind to the pentapeptide of lipid II (Fig. 3d) and therefore, unlike vancomycin, is not sensitive to variations in the pentapeptide composition^32^. This provides a rationale for the ability of teixobactin to avoid development of resistance^4,34^. Of note, the tight interaction between End10 and MurNAc constrains the flexibility of the pentapeptide (Fig. 3f). Therefore, the pentapeptide is more rigid in the complex with teixobactin than with R4L10-teixobactin, which we show with scalar^35^ ssNMR experiments (Extended Data Fig. 7c). The obstruction of the conformational space of the pentapeptide by teixobactin might hamper the recognition of lipid II by transglycosylases of the cell wall biosynthesis that bind to the pentapeptide^36^.

**Extended Data Fig. 8:**
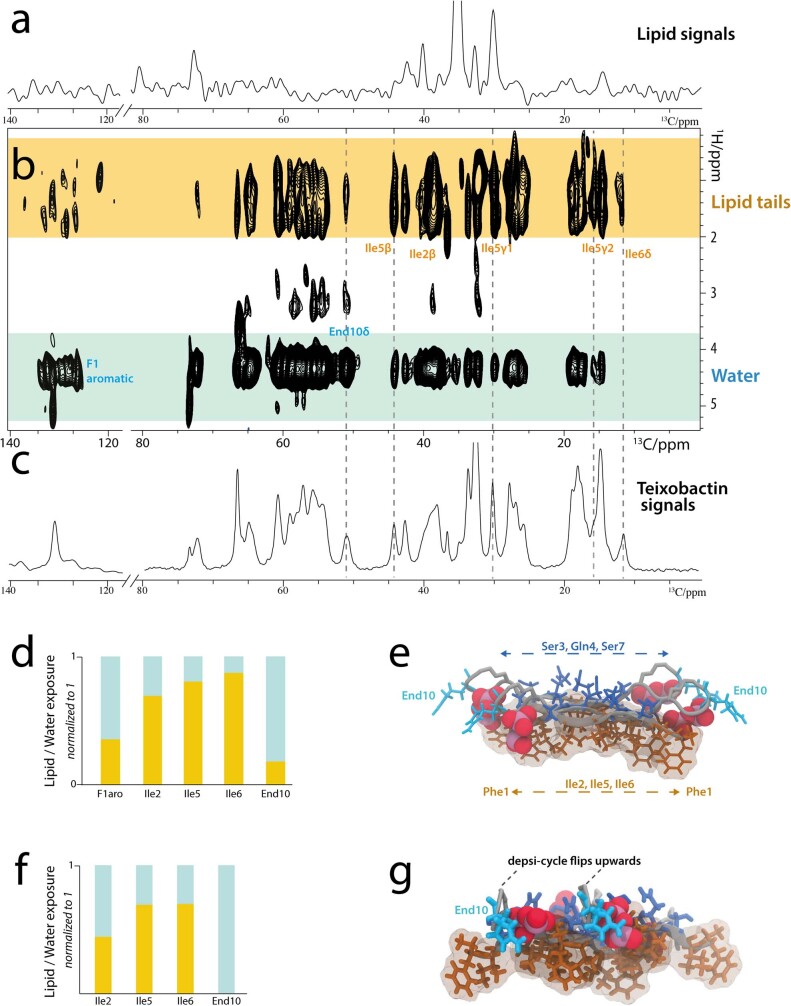
The membrane topology of the complex. a) and b) show mobility-edited (‘T_2_-filtered’) ^1^H(^1^H)^13^C ssNMR experiments^37^ of ^13^C,^15^N-teixobactin bound to Lipid II. Here, magnetization from mobile water and lipid molecules is transferred to the rigid teixobactin protons via ^1^H-^1^H mixing and eventually transferred to the ^13^C nuclei of teixobactin via a short cross polarisation step (200 μs). A 2D implementation of this experiment demonstrates that the sidechains of Ile2, Ile5, and Ile6 partition into the membrane whereas the End10 sidechain remains in the water phase. Spectra were measured at 700 MHz, 16.5 kHz MAS, and 300 K sample temperature. a) Control: 1D ^1^H(^1^H)^13^C ssNMR spectrum (10240 scans) using a T_2_-filter of 2.5 ms without transfer to teixobactin (0 ms ^1^H-^1^H mixing). All signals relate to lipids, demonstrating the effectiveness of the T_2_-filter. b) 2D ^1^H(^1^H)^13^C ssNMR spectrum using 2.5 ms T_2_ filter (8192 scans) and 5 ms ^1^H-^1^H mixing. c) ^13^C cross-polarization spectrum (200 μs contact time) of Lipid II-bound teixobactin. d) Normalized relative signal intensities of the correlations with water (blue) and lipid (brown) protons for several residues. For each residue, peak areas were integrated using Topspin 4.06. Signals that were used for the integration are annotated. e) Membrane topology visualized with the ssNMR structure. Hydrophilic (in blue) and hydrophobic (brown) residues are sharply separated above and below the β-sheet, respectively. Note that the N-terminal Phe1 at the rim of the β-sheet has higher water accessibility, potentially because of fraying and mobility effects. f) Normalized contacts to water and lipid-tails, back-calculated over the MD simulation based on our ssNMR structure. g) Membrane topology using a different structure of the calculated ssNMR ensemble. As shown in Extended Data Fig. 9a, the despi-cycle is plastic and can point up towards the water phase, bringing End10 well-above the membrane-plane. Source data

Finally, we determined the membrane topology of lipid II-bound teixobactin using a mobility-edited ssNMR experiment^37^ in which magnetization from mobile water or lipids is transferred to the rigid antibiotic. In agreement with our previous study^18^, we found that teixobactin localizes at the water–membrane interface with the hydrophobic residues Ile2, Ile5 and Ile6 partitioned in the bilayer (Extended Data Fig. 8). This is supported by a calculated^38^ partition coefficient of 5.57 for the three hydrophobic sidechains of the N-terminal tail. Together, our data provide a comprehensive picture of the structure and topology of the complex.

## Structure of the complex

Next, we calculated the complex structure using four teixobactin and four lipid II (4 × 4) molecules, which provides a better description of intermolecular interactions than a dimeric (2 × 2) arrangement. Calculations were performed with HADDOCK2.4 (ref. ^39^) and were based on intermolecular teixobactin–teixobactin and interfacial teixobactin–lipid II ssNMR distance restraints, as well as dihedral ssNMR restraints. We obtained 15 unambiguous interfacial contacts with the MurNAc sugar that define the interface precisely.

**Fig. 4:**
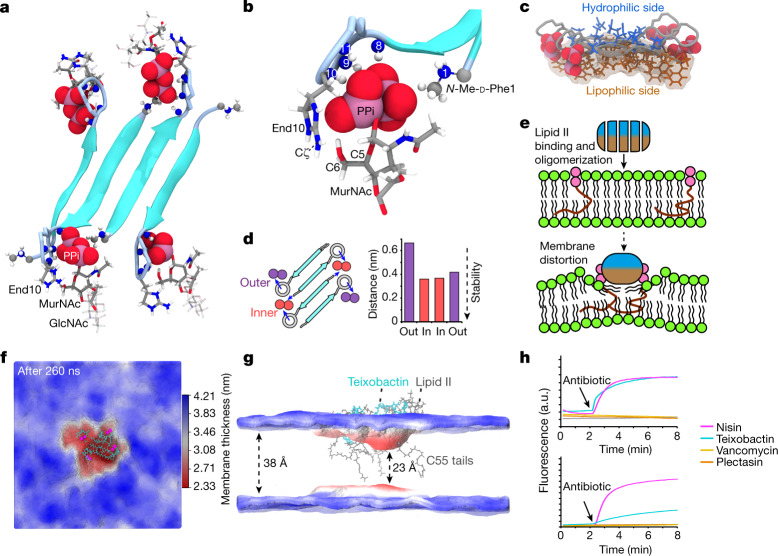
Teixobactin–membrane interaction. **a**, High-resolution ssNMR structure of the teixobactin–lipid II complex. **b**, Zoomed in view of the complex interface. The backbone amino-protons of the depsi-cycle of teixobactin, the End10 sidechain and the N terminus of an adjacent teixobactin coordinate the lipid II PPi group. In addition, End10 interacts with the MurNAc sugar via hydrogen bonds. Blue spheres represent backbone nitrogens; numbers indicate the residue numbers. **c**, Membrane topology: lipophilic and hydrophilic residues are sharply separated. **d**, Oligomerization enhances complex stability: molecular dynamics simulations show that inner lipid II molecules (red) are more stably bound than outer molecules (purple). The plot shows the distance between the centre of mass of the PPi group and the centre of mass of amino-protons of the depsi-cycle averaged over the last 200 ns of two molecular dynamics simulations. Source data are provided as a Source Data file. **e**, Upon lipid II-induced oligomerization, the hydrophobic side of teixobactin faces the membrane surface, which displaces the polar lipid headgroups and concentrates non-lamellar lipid II tails, causing membrane distortions. **f**,**g**, Membrane thickness obtained from molecular dynamics simulations, averaged over the last 50 ns. The membrane is thinner at the site of the complex. The red and blue colours show membrane thickness minima and maxima, respectively. **h**, Bacterial assays with *Staphylococcus simulans* were used to study membrane depolarization (DiSC; upper panel) and membrane damage (Sytox; lower panel) caused by teixobactin, nisin, plectasin, vancomycin and *sans* antibiotic. Arrows indicate the addition of antibiotics. Source data are provided as a Source Data file. Source data

**Extended Data Fig. 9:**
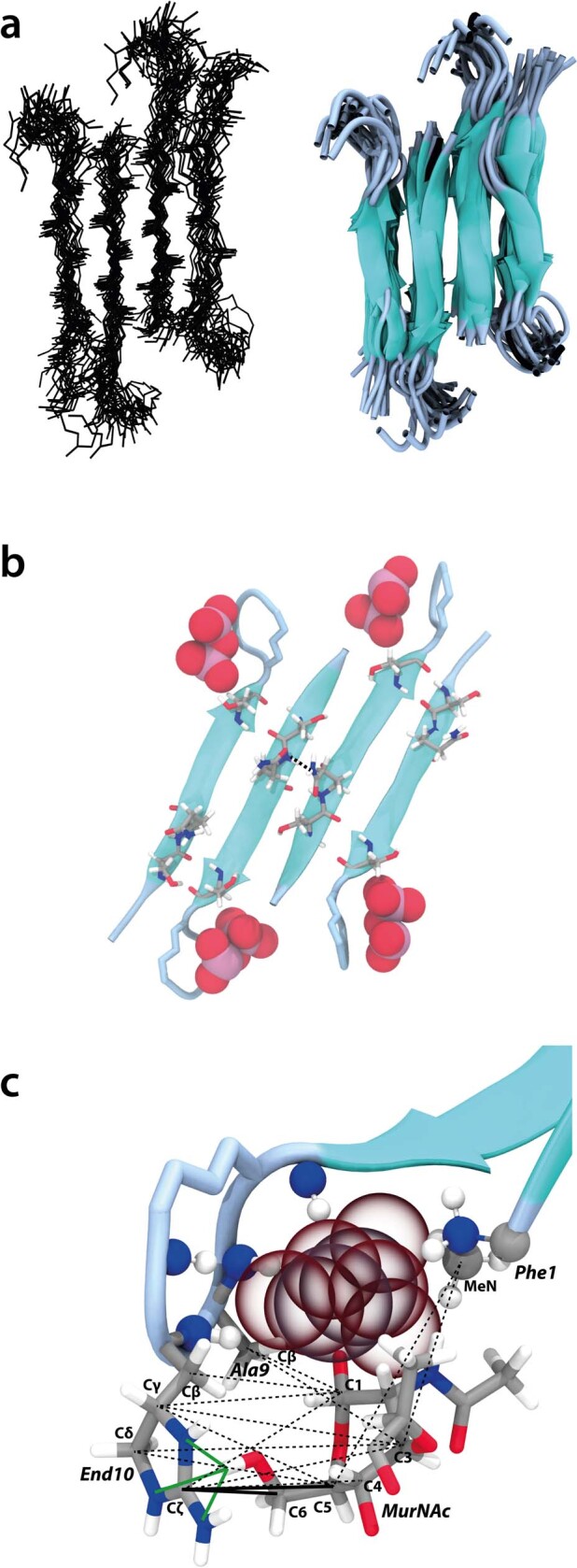
Structures of the drug in the complex superimpose well. a) Superposition of 25 structures (lines and cartoon representations) of four teixobactin bound to four Lipid II molecules (not shown). The average backbone RMSD is 2.3 +/− 0.6 Å (from the average structure). We calculated the structure of four teixobactin and four (4 x 4) Lipid II (quartet) instead of a 2 x 2 unit (dimer) to obtain a better description of the intermolecular interactions. In a dimer, half of the backbone hydrogen bonds cannot form, and the repetitive interactions between sidechains along the fibril axis are truncated. The description is improved in a quartet, in which intermolecular interactions are complete for the inner dimer, which is the reason why the inner teixobactin molecules are better defined than the outer ones. b) The network of hydrophilic residues on the water-exposed side creates opportunities for hydrogen bonding between the sidechains of D-Gln4. Moreover, MD simulations suggest that the hydroxyl-group of Ser7 favourably interacts with the Lipid II PPi group. c) Green lines show hydrogen bonds and favourable electrostatics between End10 and MurNAcC6OH. The unambiguous, short ssNMR contacts of End10Cζ with MurNAcC5 and MurNAcC6 are highlighted with thick continuous lines. Distances in the NMR structure: Ala9Cβ – MurNAcC1 = 4.0 Å. Ala9Cβ – MurNAcC3 = 6.4 Å. End10Cζ – MurNAcC6 = 4.1 Å. End10Cζ – MurNAcC5 = 5.2 Å. End10Cζ – MurNAcC4 = 5.1 Å. End10Cζ – MurNAcC3 = 6.4 Å. End10Cζ – MurNAcC1 = 6.6 Å. End10Cδ – MurNAcC5 = 5.7 Å. End10Cγ – MurNAcC5 = 5.9 Å. End10Cγ – MurNAcC3 = 7.0 Å. End10Cγ – MurNAcC1 = 6.6 Å. End10Cβ – MurNAcC1 = 5.6 Å. Phe1MeN – MurNAcC3 = 6.0 Å. Phe1MeN – MurNAcC5 = 7.2 Å. End10Nδ – MurNAcC6OH = 2.6 Å (hydrogen bond/electrostatic interaction). End10Nε – MurNAcC6OH = 2.2 Å (hydrogen bond/electrostatic interaction). End10Nζ – MurNAcC6OH = 3.2 Å (hydrogen bond/electrostatic interaction).

We obtained a well-resolved ensemble (2.3 ± 0.6 Å average backbone RMSD (root-mean square deviation) for teixobactin) that shows an antiparallel teixobactin β-sheet that could elongate into fibrils (Fig. 4a and Extended Data Fig. 9a), in agreement with the HS-AFM data. The β-sheet is out of register, creating the space to accommodate lipid II between the termini of the neighbouring β-strands. The hydrophilic (Ser3, Gln4 and Ser7) and hydrophobic (Phe1, Ile2, Ile5 and Ile6) residues of teixobactin are sharply divided above and below the β-sheet, respectively (Fig. 4c), which firmly anchors the complex in the membrane and stabilizes the β-sheet by intermolecular hydrogen bonds (Extended Data Fig. 9b). This separation is enabled by the defined sequence of d-amino and l-amino acids in teixobactin, explaining why changing this sequence drastically curbs the activity^15,18^.

The interface between teixobactin and the PPi–MurNAc group of lipid II is well defined. In the oligomeric complex, PPi is tightly coordinated by the amino-protons of the depsi-cycle of teixobactin and the cationic N terminus of the neighbouring teixobactin strand. This dual coordination of lipid II by adjacent teixobactin molecules improves the stability of the interface (Fig. 4b), explaining the functional importance of the cationic N terminus^20,21^. The complex shows a specific teixobactin–MurNAc interaction that is governed by End10, in agreement with our ssNMR and isothermal titration calorimetry data. At the interface, End10 is poised to form hydrogen bonds with the C6-OH group of MurNAc in immediate proximity, which agrees with the short-distance ssNMR contacts that we observed between End10Cζ–MurNAcC6 and End10Cζ–MurNAcC5 (Extended Data Fig. 9c). Moreover, the cationic End10 is close to the anionic PPi, causing favourable electrostatics. The void between the β-strands is closed by the acetyl group of MurNAc, which engages in hydrophobic interactions with the methyl group of Phe1, corroborated by ssNMR contacts of the methyl group to MurNAcC1 and MurNAcC3. Neither GlcNAc nor the pentapeptide of lipid II is directly engaged in any interactions with natural teixobactin. Notably, GlcNAc consistently points downwards in the complex structure (Fig. 4a), suggesting that it interacts with the membrane headgroup region.

Together, these data show that natural teixobactin tightly and specifically binds to the invariable PPi–MurNAc part that is present in several cognate cell wall precursors such as lipid I, lipid II or lipid III (precursor of wall teichoic acid), whereas it shuns the pentapeptide that varies across bacterial strains^32^. Therefore, natural teixobactin maximizes its target spectrum while minimizing the likelihood of resistance development. Notably, the specific interface between natural teixobactin and PPi–MurNAc contrasts with the fuzzy interface of R4L10-teixobactin, where the hydrophobic residues of the depsi-cycle do not favourably interact with MurNAc.

## A hydrophobic wedge splays the membrane

We next probed the stability of the complex structure with molecular dynamics simulations. From simulations, we also thought to gauge how the complex interacts with the membrane. We equilibrated the complex in a hydrated DOPC membrane and then freely evolved the system over 260 ns. The complex remained stable over the entire simulation, which vouches for the quality of the ssNMR structure. The simulation demonstrates the importance of oligomer formation for a stable teixobactin–lipid II interaction. The inner lipid II molecules, which are dual-coordinated by adjacent teixobactin molecules, were more tightly bound than the outer lipid II molecules, which are only mono-coordinated by one teixobactin (Fig. 4d). This behaviour was confirmed in another molecular dynamics simulation of equal duration.

**Extended Data Fig. 10:**
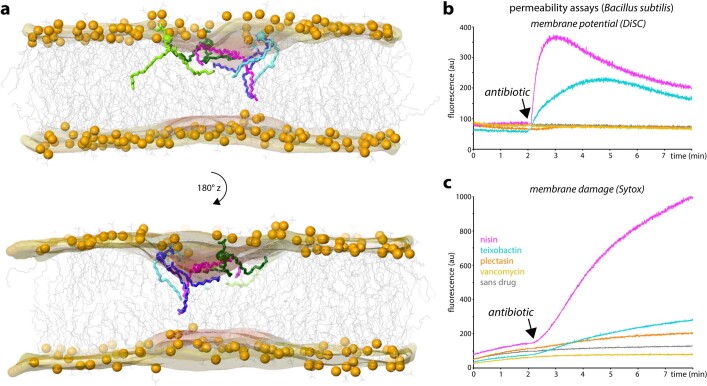
Oligomeric teixobactin – Lipid II complexes thin the membrane by displacing lipids. a) MD simulation of the teixobactin – Lipid II complex after 248 ns of free evolution. The membrane surface is shown in light orange, and the phosphate-atoms of phospholipids are shown as orange spheres. The complex is not shown for clarity but is localised centrally on the upper membrane leaflet where membrane distortions are most pronounced. Lipids below the complex, for which the tails show major distortions, are shown in stick representation and in different colours. Polar headgroups of distorted lipids avoid the hydrophobic underbelly of the teixobactin β-sheet, which causes lipid tails to orient almost parallel to the membrane plane, resulting in a thinning of the membrane. The high concentration of non-lamellar C55-tails of Lipid II (not shown) likely also contributes to membrane perturbations at the complex site. Permeability assays with *Bacillus subtilis*: Bacterial assays with *Bacillus subtilis* were used to study b) membrane depolarization and c) membrane damage caused by teixobactin (in blue), nisin (magenta), plectasin (orange), vancomycin (yellow), and *sans* antibiotic (gray). Source data are provided as a Source Data file. Source data

The complex caused a pronounced perturbation of the membrane, which manifests itself in a sharply reduced membrane thickness (2.33 ± 0.23 nm at the complex site compared to 3.82 ± 0.23 nm for the unperturbed membrane) (Fig. 4e–g). Membrane perturbations are caused by the very hydrophobic surface of the teixobacin β-sheet that faces the membrane. The hydrophobic β-sheet surface acts like a wedge that pushes the polar lipid headgroups underneath aside, causing lipids to bend almost parallel to the membrane plane (Extended Data Fig. 10a). The membrane order is additionally perturbed by the concerted action of the highly non-lamellar C55 tails of lipid II, which reach deep into the lipid layer on the opposite side to the complex. It appears that teixobactin hijacks lipid II to help disrupt the membrane. These membrane perturbations are in agreement with the membrane deformation that we detected with microscopy and ssNMR.

In much larger teixobactin–lipid II oligomers that we observe experimentally, the perturbations must certainly be more pronounced than in the simulated building block. We hence hypothesized that the membrane perturbations caused by the supramolecular structure could contribute to the antimicrobial action of teixobactin. Addition of teixobactin to Gram-positive bacteria showed a sharp decrease in the membrane potential^40^ (Fig. 4h and Extended Data Fig. 10b,c). Membrane depolarization was also observed for nisin, a well-known pore former^41^, but not for the lipid II binders plectasin^42^ or vancomycin^43^. Although less pronounced than for nisin, teixobactin also showed clear effects with Sytox green^44^, a high-affinity DNA stain that can only traverse damaged membranes. Such effects were again absent for plectasin or vancomycin. Membrane defects using intact bacteria were observed within 10 min, that is, a timescale that coincides with the emergence of clusters in bacteria and of fibrils in HS-AFM studies. Together, ssNMR, HS-AFM, confocal microscopy and computer simulations demonstrate that the formation of teixobactin–lipid II fibrillar structures causes membrane defects that contribute to the killing mechanism of teixobactin. Action against the membrane explains why teixobactin is more effective in killing bacteria than vancomycin, which binds to the pentapeptide of lipid II^4^.

## Discussion

The mechanistic data on teixobactin–lipid II binding uncovered in this study illuminate the unique mode of action of this unusual antibiotic. Settling on the surface of the bacterial membrane, the hydrophobic Ile and d-*allo*-Ile residues anchor it to the membrane. From the 3D space of the external medium, teixobactin preferentially partitions at the 2D water–membrane interface. The structure of the teixobactin–lipid II complex resolved by ssNMR shows that the enduracididine sidechain tightly binds PPi and the MurNAc sugar. Enduracididine forms strong and selective interactions with the MurNAc hexose but would not be able to form a similar interaction with a sterically different pentose sugar, which explains why teixobactin avoids binding to the PPi-(deoxy)ribose moiety of purine nucleosides that are commonly present in the bacterial environment. Intermittent localization of l-amino and d-amino acids favours the formation of an antiparallel β-sheet interaction between two adjacent teixobactin molecules. The N terminus of teixobactin coordinates PPi of lipid II, contributing to target capture. The β-sheet of teixobactins bound to lipid II grows into a supramolecular fibrillar structure. The formation of this structure is probably irreversible, acting as a sink that further concentrates teixobactin at its site of action. Apart from efficiently and potently sequestering lipid II, the supramolecular structure displaces phospholipids, thinning the membrane and compromising its integrity. The hydrophobic residues anchoring teixobactin to the membrane are short, and unlikely to cause damage when present on isolated molecules. Combined in a supramolecular structure, however, this produces a concentrated hydrophobic patch. Similarly, and perhaps more importantly, concentrating the long C55 hydrophobic tails of lipid II within this hydrophobic patch contributes to the damage, causing ion leaks and a drop in the membrane potential. Teixobactin corrupts its target by converting it into a membrane disruptor, and in this regard, joins a select group of other target-corrupting natural products such as aminoglycosides that cause mistranslation^45^ and acyldepsipeptide^46,47^, an activator of proteolysis. Conventional membrane-acting antibiotics such as nisin form pores or carry a large hydrophobic moiety that is sufficient to damage the membrane of both bacteria and eukaryotic cells, causing toxicity. Producing membrane-acting antibiotics that do not harm human cells has thus far proven to be an elusive goal^3^. The problem of selectivity is elegantly solved in teixobactin by disrupting the membrane only if a supramolecular structure forms, and it only forms if the membrane carries lipid II. The formation of the supramolecular structure by teixobactin is a striking departure from the conventional one ligand–one target paradigm. The dual action of teixobactin in inhibiting peptidoglycan synthesis and disrupting the membrane provides for an effective attack on the bacterial cell envelope, whereas binding to an immutable target produces an antibiotic free of detectable resistance.

## Methods

### Materials

Phospholipids 1,2-dioleoyl-*sn*-glycero-3-phosphocholine (C18:1; DOPC) was purchased from Avanti Polar Lipids, Inc.

### Sample production

#### Production of teixobactin

Uniformly ^13^C,^15^N-labelled teixobactin was produced by fermentation in its native host *Eleftheria terrae*. In brief, the isolate was grown from a freezer stock on SMSR4 agar (0.125 g casein digest, 0.1 g potato starch, 1 g casamino acids, 1 g d-glucose, 0.1 g yeast extract, 0.3 g proline, 1 g MgCl_2_-6H_2_O, 0.4 g CaCl_2_-2H_2_O, 0.02 g K_2_SO_4_, 0.56 g TES free acid (2-[[1,3-dihydroxy-2-(hydroxymethyl)propan-2-yl]amino]ethanesulfonic acid) per 1 l dIH_2_O, pH to 7 with KOH and 20 g of bacto agar autoclaved at 121 °C for 45 min) for 9 days at 28 °C. Of biomass, 1 cm^2^ was transferred to 20 ml of modified Celtone-RAZDAZ (10 g d-glucose U-^13^C6, #CLM-1396, 1.1 g Celtone Base Powder ^13^C;^15^N, #CGM-1030P-CN, 0.5 g l-isoleucine ^13^C6; ^15^N, #CNLM-561-H, 10 g MgCl_2_-6H_2_O, 4 g CaCl_2_-2H_2_O, 0.2 g K_2_SO_4_, 5.6 g TES free acid per litre, pH to 7 with KOH and autoclaved at 121 °C for 45 min) and grown at 28 °C for 4 days. All labelled material was purchased from Cambridge Isotope Laboratories. Of the grown liquid culture, 20 ml was transferred to 1 l of modified Celtone-RAZDAZ and grown at 28 °C for 6 days. Biomass was harvested by centrifugation at 4,200 r.p.m. and the pellet was extracted with 1 l of 50% aqueous acetonitrile and the suspension again centrifuged for 30 min. The acetonitrile was removed from the supernatant by rotary evaporation under reduced pressure until only water remained. The mixture was then extracted twice with 1 l of *n*-BuOH. The organic layer was transferred to a round bottom flask and the *n*-BuOH was removed by rotary evaporation under reduced pressure. The resulting yellow solid was dissolved in DMSO and subjected to preparatory HPLC (high-performance liquid chromatography) (solid phase:C18, mobile phase:H2O/MeCN/0.1% TFA). The fractions containing teixobactin were then pooled and the acetonitrile was removed by rotary evaporation under reduced pressure. The remaining aqueous mixture was then lyophilized to leave a white powder (trifluoroacetate salt). In addition, a quasi-molecular ion peak of *m*/*z* 1,315.8683 for ^13^C_58_H_96_^15^N_15_O_15_ [M+H]^+^ (calculated 1,315.8706 for ^13^C_58_H_96_^15^N_15_O_15_) was determined by high-resolution electrospray ionization mass spectroscopy, confirming ^13^C_58_H_95_^15^N_15_O_15_ as the molecular formula.

#### Synthesis and purification of lipid II

Lipid II was produced according to published methods based on enzymatic lipid reconstitution using the lipid II precursors UDP-GlcNAc, UDP-MurNAc-pentapeptide and polyisoprenolphosphate as substrates^24^. Lysine-form UDP-MurNAc-pentapeptide was extracted from *S. simulans* 22. ^13^C,^15^N-labelled UDP-GlcNAc and UDP-MurNAc-pentapeptide (lysine form) were extracted from *S. simulans* 22 grown in [^13^C/^15^N]-labelled rich medium (Silantes) and supplemented with [U-^13^C]-d-glucose and [^15^N]-NH_4_Cl^19^. Polyisoprenolphosphate was synthesized via phosphorylation of polyisoprenol obtained from *Laurus nobilis*^48^. The headgroup precursors were extracted from bacteria and polyisoprenol was extracted from leaves as previously described^49^. After synthesis, lipid II was extracted with 2:1 BuOH:(Pyr/acetate; 6 M) and then purified with a DEAE cellulose resin using a salt gradient of 0–600 mM NH_4_HCO_3_ with 2:3:1 CHCl_3_:MeOH:[H_2_O + salt]. Fractions containing pure lipid II were pooled, dried and dissolved in 2:1 chloroform/methanol. The concentration of lipid II was estimated through an inorganic phosphate determination^50^.

#### ssNMR sample preparation

Multi-lamellar vesicles of DOPC doped with 4 mol% lysine-lipid II in buffer (40 mM Na_2_PO_4_ and 25 mM NaCl, pH 7.2) were collected by centrifugation (60,000*g*) and loaded into ssNMR rotors. For 3.2-mm rotors, we used 800 nmol of teixobactin with unlabelled lipid II, whereas we used 400 nmol with labelled lipid II. For 1.3-mm rotors, samples contained 200 nmol of antibiotic for unlabelled lipid II.

### ssNMR spectroscopy

^1^H-detected ssNMR experiments were performed at 60 kHz magic angle spinning (MAS) using magnetic fields of 700 and 950 MHz (^1^H frequency). 3D CαNH and CONH experiments^51^ for the sequential assignment of teixobactin were performed with dipolar transfer steps using low-power PISSARRO^52^ decoupling in all dimensions. ^1^H-detected ^15^N T_1rho_ relaxation experiments^18,51^ were acquired with a ^15^N spin lock-field of 18 kHz and spin-lock durations of 0, 10, 20, 40, 70 and 100 ms. T_1rho_ trajectories were fit to single exponentials. 2D CC experiments were acquired with PARISxy^33,53^ recoupling (*m* = 1) at 950 MHz magnetic field and 18 kHz MAS. A 2D CαN experiment was acquired at 700 MHz, 12 kHz MAS and 5 ms N to C cross-polarization transfer time. To characterize lipid II-bound teixobactin, we used CC magnetization transfer times of 50 and 600 ms. To probe interfacial contacts between ^13^C,^15^N-teixobactin and ^13^C,^15^N-lipid II, we used CC magnetization transfer times of 50, 150 and 300 ms. The scalar TOBSY^35^ experiment was acquired at 700 MHz using 8 kHz MAS with 6 ms CC mixing time. The mobility edited H(H)C experiment^25^ was measured at 700 MHz with 16.5 kHz MAS at a temperature of 300 K using a T_2_ relaxation filter of 2.5 ms. 1D MAS ^31^P experiments were acquired at 500 MHz magnetic field and 12 kHz MAS. 2D HP experiments were acquired at 800 MHz and 60 kHz MAS using 1 and 2 ms ^1^H to ^31^P cross-polarization contact time. Static ^31^P ssNMR experiments were acquired at 500 MHz magnetic field without sample spinning. Note that the phosphorus nuclei of lipids give rise to an anisotropic powder pattern signal, whose shape depends on the orientation of lipid headgroups^25^. Further experimental details of ssNMR experiments are given in the Supplementary Information.

### Fluorescence microscopy

#### GUVs preparation

We used a self-assembled GUV cell, aligned with two titanium electrodes in a closed Teflon chamber (volume = 500 μl). Of 0.5 mM DOPC doped with Atto 550-labelled lipid II (0.1 mol%), 1 μl was brushed on the titanium electrodes. The GUV cell was dried under vacuum. Next, the chamber was filled with 350 μl 0.1 M sucrose solution, the electrodes dipped in and connected to a power supply of a sine wave (2.5 V; 10 Hz; 90 min). Each microscopy slide (m-slide 8 well, Ibidi) was incubated with 350 μl BSA solution (1 mg ml^−1^) for 1 h. To detach the GUVs, the power supply was changed to square wave (2 V; 2 Hz; 15 min). The slides were washed once with water and 0.1 M glucose solution. The slides were immersed in 300 μl of 0.1 M glucose solution to which 50 μl of GUVs was added. These were incubated for 3 h with 1 μM teixobactin and later observed under a Zeiss LSM 880 confocal microscope. GUVs were imaged using Zeiss LSM 880 with ×63/1.2 NA glycerol and ×100/1.2 NA oil objective lenses. The Atto 550 label appeared red upon excitation by the 560-nm laser. The brightfield was used for detection and location of the GUVs and to observe their shape. Zeiss Zen Black software was used for the analysis of the images.

#### Bacterial imaging

*B. megaterium* was grown overnight at 37 °C in LB media. Secondary culture was grown for 3 h until the OD_600_ = 0.3 was reached. Of cells, 500 μl were centrifuged at 3,000*g* for 5 min. The supernatant was discarded, and the cells were resuspended in 200 μl solution from a 1 μg ml^−1^ stock of the fluorescent analogue^26^ Lys(Bodipy FL)_10_-teixobactin. The cells were allowed to incubate for the desired timepoints (1 min, 15 min and 45 min) at 37 °C. After incubation, they were centrifuged and washed with buffer (100 mM Na_2_HPO_4_ and 18 mM KH_2_PO_4_, pH 7.4) three times. For fixing the cells, they were resuspended in a 4% formalin and allowed to incubate at 37 °C for 10 min. They were washed once again with the buffer and resuspended in 200 μl of buffer. Of the stained and fixed cells, 50 μl were then pipetted onto the agarose beds and covered with coverslip. The bacterial coverslips were imaged using Zeiss LSM 700 with a ×100/1.2 NA oil objective lens. Lys(Bodipy FL)_10_-teixobactin was excited using a 488-nm laser. A z-stack containing 15 planes at a 0.56-μm interval was acquired with 0.1-μm pixel size, and maximum intensity projections were made for analysis and display. Icy software’s Spot detector was used to analyse the images and calculate the average intensity of the clusters in all images^54^.

### Isothermal titration calorimetry

For isothermal titration calorimetry (ITC) measurements large unilamellar vesicles (LUVs) containing lysine-lipid II were prepared by incorporating 2 mol% of lysine-lipid II in DOPC from the stock solution. The lipids were dried under a nitrogen stream and hydrated with buffer (20 mM HEPES and 50 mM NaCl, pH 7) to a lipid-phosphate concentration of 20 mM. Finally, unilamellar vesicles were obtained after ten rounds of extrusion through 200-nm membrane filters (Whatman Nuclepore, Track-Etch Membranes). ITC experiments were performed with the Affinity ITC (TA Instruments-Waters LLC) to determine interaction between LUVs and teixobactin. Teixobactin was diluted in the buffer, to a final concentration of 30 μM. The samples were degassed before use. The chamber was filled with 177 μl of teixobactin, and the LUVs were titrated into the chamber at a rate of 1.96 ml per 150 s with a constant syringe stirring rate of 125 r.p.m. The number of injections was 23. Experiments were performed at 37 °C and analysed using the Nano Analyze Software (TA instruments-Water LLC). All experiments were performed in triplicates. Control experiments were performed with lipid II-free DOPC LUVs. The independent model was used to determine the interaction between teixobactin and lipid II. ITC data of R4L10-teixobactin were previously published^18^.

### Fluorescence spectroscopy

For fluorescence spectroscopy, DOPC LUVs containing 0.5 mol% of pyrene-labelled lipid II in buffer (10 mM Tris-Cl and 100 mM NaCl, pH 8.0) were prepared as described above. All fluorescence experiments were performed with a Cary Eclipse (FL0904M005) fluorometer. All samples (1.0 ml) were continuously stirred in a 10 × 4-mm quartz cuvette and kept at 20 °C. Teixobactin was titrated to the LUVs. Pyrene fluorescence was followed with spectral recordings between 360 and 550 nm (*λ*_ex_350 nm, bandwidth 5 nm). The emission at 380 and 495 nm was recorded and averaged over 50 s, to obtain the values for the monomer and excimer intensity, respectively, to determine the excimer to monomer ratio for all conditions.

### HS-AFM imaging

The HS-AFM images were acquired in amplitude modulation tapping mode in liquid using a high-speed atomic force microscope (RIBM). Short cantilevers (approximately 7 μm) with a nominal spring constant of 0.15 N m^−1^ were used (USC-F1.2-k0.15, NanoWorld). A minimal imaging force was applied by using a small set-point amplitude of 0.8 nm (for a 1 nm free amplitude). The HS-AFM results showing the assembly of teixobactin filaments and membrane deformation were obtained from imaging of supported lipid bilayers on mica. The lipid bilayer was obtained by incubating LUVs containing DOPC and lipid II (prepared as mentioned above) on top of a freshly cleaved mica for 20–30 min. After the incubation period, the mica was cleaned gently using recording buffer (10 mM Tris-Cl and 100 mM NaCl, pH 8.0). Imaging was started on the lipid bilayer surface in recording buffer. Next, a concentrated teixobactin solution was added to reach the desired final teixobactin concentration in the AFM liquid chamber of 40 µl. Images were primarily processed using built-in scripts (RIBM) in Igor Pro (Wavemetrics) and analysed using ImageJ software. The images or videos were corrected minimally for tilt, drift and contrast. Unless otherwise mentioned, the times reported in AFM images are relative to the addition of teixobactin into the imaging chamber. Image acquisition rate varies from 0.5 frames per second to 2 frames per seconds (see Fig. 2, Extended Data Figs. 5, 6,  or legends of Supplementary Videos 1 and 2), and the line rate varies from 150 lines per second to 400 lines per second. Control experiments with conventional AFM (JPK Nanowizard) supported the HS-AFM measurements as a similar height of the individual fibrils and their sheets on the membrane was observed. Stated errors are standard deviation.

### Permeabilization assay

The bacterial cultures were grown overnight at 30 °C in TSB media for *S. simulans* and at 37 °C in LB medium for *Bacillus subtilis*. Secondary cultures were grown for 3 h until OD_600_ = 0.5 was reached. The bacterial cells were then centrifuged at 1,500*g* for 10 min at 4 °C and washed twice with 10 ml of buffer (10 mM Tris, 100 mM NaCl, 1 mM MgCl_2_ and 0.5% glucose, pH 7.2). The bacterial cells were resuspended to an OD_600_ = 10 in the buffer and used for the experiment. All permeability experiments were performed with a Cary Eclipse (FL0904M005) fluorometer. All samples (1.0 ml) were continuously stirred in a 10 × 4-mm quartz cuvette and kept at 20 °C. For the assay, 1 μl of the bacterial suspension was added to 1 ml of buffer. For the ion leakage assays, 1 μl of the DiSC-2 probe from a 1 mM stock was added to the cuvette and the fluorescence was measured between a wavelength of 650 nm and 670 nm (bandwidth of 5 mm) for 2 min before the addition of the antibiotic and 6 min after. For the Sytox green leakage assays, 1 μl of the Sytox green probe from a 0.25 mM stock was added to the cuvette and the fluorescence was measured between a wavelength of 500 nm and 520 nm (bandwidth of 5 mm) for 2 min before the addition of the antibiotic and 6 min after. All experiments were performed in triplicates. The concentrations of antibiotics used are 10 nM nisin (1× MIC), 10 μM vancomycin (10× MIC) and 0.5 μM plectasin/teixobactin for *S. simulans* (1× MIC) and 0.2 μM plectasin/teixobactin for *B. subtilis* (10× MIC).

### Structure calculations

#### Parametrization of teixobactin

Parameters and topology were based on our work on R4L10-teixobactin^18^, substituted with d-glutamine at position 4 and l-*allo*-enduracididine at position 10. Parameters for l-*allo*-enduracididine were based on l-arginine, in which the guanidinium group was cyclized with ring geometry as in 2-keto-enduracididine in Protein Data Bank (PDB) 4JME^55^. A monomeric teixobactin starting model for HADDOCK structure calculation was then generated in CNS^56^, using only chemical-shift-derived restraints^57^. Parameters for lipid II were taken from ref. ^58^.

#### Structure calculation protocol

We used HADDOCK version 2.4 (ref. ^39^) for the structure calculations. An eight-body docking (four lipid II and four teixobactin molecules) was performed using ssNMR-derived distance and dihedral restraints. Seven thousand models were generated in the rigid-body docking stage of HADDOCK, of which the best-scoring 500 were subjected to the flexible refinement protocol of HADDOCK. The resulting models were energy minimized. Default HADDOCK settings were used except for doubling the weight of the distance restraints during all stages of the structure calculation. The final models were further filtered based on the topological requirements (that is, the lipid tails of all lipid II molecules must point in the same direction as the membrane-anchoring residues Ile2, Ile5 and Ile6). This resulted in a final ensemble of 25 structures.

#### Analysis of calculated structures

Structural and violation statistics of the final 25 structures are discussed in detail in the Supplementary Information. The average backbone RMSD (from the average structure) of the 25 teixobactin molecules in the complex was 2.3 ± 0.6 Å.

### Molecular dynamics simulations

Molecular dynamics calculations were performed with GROMACS, version 4.6.3 using the g54a7 forcefield^59^. We simulated the ssNMR structure of four teixobactin molecules in complex with four lipid II molecules in a hydrated DOPC membrane. The truncated lipid II tail used for the ssNMR structure was manually elongated to C55 tails by transferring coordinates from ref. ^60^. The topologies for natural teixobactin and lipid II were generated using ATB^61^. The charges on the PPi group were adapted to those in ref. ^58^. For the starting system, the complex was placed approximately 0.5 nm (in reference to the teixobactin molecules) above a pre-equilibrated DOPC bilayer^62^ (extended to 512 lipids) and ten lipids were removed to accommodate the long lipid II tails. The box (dimensions 12.81 × 12.81 × 10 nm) was then rehydrated and the system electrostatically neutralized (total atom number of 120,295). After minimization, the system was equilibrated at 300 K for 1 ns in an NVT ensemble (fixed number of atoms, N, a fixed volume, V, and a fixed temperature, T) using a V-rescale thermostat with a coupling constant of 0.1 ps and a 2-fs time step with strong position restrains (force constant of 10,000 kJ mol^−1^ nm^−2^) on the complex. Next, the system was equilibrated for 100 ns in an NPT ensemble (fixed number of atoms, N, a fixed pressure, P, and a fixed temperature, T) with semi-isotropic pressure coupling at 1 bar using a Parrinello–Rahman barostat^63^. During this equilibration step, position restraints were gradually reduced from 1,000 to 25 kJ mol^−1^ nm^−2^. For lipid II tails, position restraints were removed to facilitate their integration into the membrane. Afterwards, the system was freely evolved in two independent simulations for 287 and 267 ns without applying ssNMR distance restraints. In one of the two simulations, chemical-shift-derived dihedral restraints^57^ were applied to residues 2–6 of the teixobactin molecules with a constant of force of 100 kJ mol^−1^ nm^−2^.

Average atom–atom distances in the ensemble (see Supplementary Tables 5–7) were computed with the GROMACS tool g_dist. The membrane thickness discussed in Fig. 4 was computed with g_lomepro^64^, considering the phosphorus atoms of DOPC to specify the representative lipid atoms and using a 100 × 100 grid. An additional simulation of 250 ns without teixobactin was performed to get the average thickness of the unperturbed membrane. To back-calculate distances between teixobactin and water or lipid tails, we counted contacts over the free molecular dynamics simulation (from 100 ns to the end of the simulation). Contacts were counted using the GROMACS tool g_mindist for a water or lipid tail atom within a distance of 0.5 nm.

### Reporting summary

Further information on research design is available in the Nature Research Reporting Summary linked to this paper.

## Online content

Any methods, additional references, Nature Research reporting summaries, source data, extended data, supplementary information, acknowledgements, peer review information; details of author contributions and competing interests; and statements of data and code availability are available at 10.1038/s41586-022-05019-y.

## Supplementary information

Supplementary Information This file contains Supplementary Figs. 1–3, Supplementary Tables 1–10, the Supplementary Discussion, analysis of the calculated structure, details of 2D and 3D ssNMR experiments, the LogP calculation and Supplementary References.

Reporting Summary

Supplementary Video 1 Assembly of teixobactin – Lipid II fibrils as captured by HS-AFM on a DOPC-lipid bilayer containing 1% Lipid II in presence of 800 nM teixobactin. Imaging speed 0.5 frame/s.

Supplementary Video 2 Assembly of teixobactin – Lipid II fibrils as captured by HS-AFM on a DOPC-lipid bilayer containing 1% Lipid II in presence of 1µM teixobactin. Imaging speed 2 frames/s.

Supplementary Video 3 Animation of 3D rendered images of membrane deformed by teixobactin – Lipid II fibrils shown from different angles. The z scale bar (1 nm) is relative and only refers to the depicted top part of the membrane. The 3D image frame dimensions are 265 nm in X-Y and 0.5 nm in Z.

## Data Availability

The ssNMR assignments of teixobactin and lipid II have been deposited in the Biological Magnetic Resonance Data Bank (accession number 50938). The PDB structure of the complex has been deposited in the PDB database (PDB code 7QGV). Experimental ssNMR raw data have been deposited in an open repository, Zenondo (10.5281/zenodo.6549335). The source data underlying Figs. 1c,h, 2d, 3e and 4d,h and Extended Data Figs. 2, 5, 6, 8d,f and 10b,c are provided as a Source Data file. Source data are provided with this paper.

## Source data

Source Data Figs. 1–4, Extended Data Figs. 2, 5, 6, 8 and 10
