# Supplementary material for: Teixobactin kills bacteria by a two-pronged attack on the cell envelope
Source: Nature. 2022 Aug 3;608(7922):390–6. doi: 10.1038/s41586-022-05019-y (PMC9365693; doi:10.1038/s41586-022-05019-y)
Supplement: Supplementary file 1 — This file contains Supplementary Figs. 1–3, Supplementary Tables 1–10, the Supplementary Discussion, analysis of the calculated structure, details of 2D and 3D ssNMR experiments, the LogP calculation and Supplementary References. [file 41586_2022_5019_MOESM1_ESM.docx]

**SUPPLEMENTARY INFORMATION**

**Teixobactin kills bacteria by a two-pronged attack on the cell envelope**

Rhythm Shukla^1,2^, Francesca Lavore^1^, Sourav Maity^3^, Maik G.N. Derks^1,2^, Chelsea R. Jones^4^, Bram J.A. Vermeulen^1^, Adéla Melcrova^3^, Michael A. Morris^4^, Lea Marie Becker^1^, Xiaoqi Wang^2^, Raj Kumar^1^, João Medeiros-Silva^1^, Roy A.M. van Beekveld^1^, Alexandre M.J.J. Bonvin^1^, Joseph H. Lorent^2^, Moreno Lelli^5,6^, James S. Nowick^4^, Harold D. MacGillavry^7^, Aaron J. Peoples^8^, Amy L. Spoering^8^, Losee L. Ling^8^, Dallas E. Hughes^8^, Wouter H. Roos^3^, Eefjan Breukink^2^, Kim Lewis^9^, and Markus Weingarth^1,^*

*^1^NMR Spectroscopy, Bijvoet Centre for Biomolecular Research, Department of Chemistry, Faculty of Science, Utrecht University, The Netherlands*

*^2^Membrane Biochemistry and Biophysics, Bijvoet Centre for Biomolecular Research, Department of Chemistry, Faculty of Science, Utrecht University, The Netherlands*

*^3^Moleculaire Biofysica, Zernike Instituut, Rijksuniversiteit Groningen, Groningen, The Netherlands*

*^4^Department of Chemistry, University of California Irvine, Irvine, CA, USA*

*^5^Magnetic Resonance Center (CERM) and Department of Chemistry ‘Ugo Schiff’, University of Florence, Sesto Fiorentino (FI), Italy*

*^6^Consorzio Interuniversitario Risonanze Magnetiche MetalloProteine (CIRMMP), Sesto Fiorentino (FI), Italy*

*^7^Cell Biology, Neurobiology and Biophysics, Department of Biology, Faculty of Science, Utrecht University, Utrecht, The Netherlands*

*^8^NovoBiotic Pharmaceuticals, Cambridge, MA, USA*

*^9^Antimicrobial Discovery Center, Northeastern University, Department of Biology, Boston, MA, USA*

**corresponding author: m.h.weingarth@uu.nl*

**Supplementary Fig. 1:**

**Annotated chemical structure of natural teixobactin.**

The chemical structure of the natural undecapeptide teixobactin. Backbone nitrogen nuclei and amino protons observed in Fig. 1b are colored in blue.

**Supplementary Table T1:**

^13^C,^15^N-labelled Lipid II, bound state, obtained from ssNMR measurements in DOPC liposomes. Assignments in ppm.

| **Sugars** | **#** | **C1** | **C2** | **C3** | **C4** | **C5** | **C6** | **C7** | **C8** | **C9** | **C10** | **C11** |
| --- | --- | --- | --- | --- | --- | --- | --- | --- | --- | --- | --- | --- |
| **MurNAc** |  | 98.9 | 57.2 | 81.7 | 74.4 | 77.6 | 62.5 | 177.0 | 25.9 | 81.3 | 179.1 | 22.1 |
| **GlcNAc** |  | 102.6 | 59.5 | 79.6 | 73.8 | 77.2 | 64.5 | 177.1 | 26.0 |  |  |  |
|  |  |  |  |  |  |  |  |  |  |  |  |  |
| **Pentapep** |  | **CO** | **Cα** | **Cβ** | **Cγ** | **Cδ** | **Cε** |  |  |  |  |  |
| **L-Ala** | **1** | 176.9 | 52.7 | 19.7 |  |  |  |  |  |  |  |  |
| **D-γ-Glu** | **2** | 179.1 | 31.5 | 30.5 | 57.4 | 181.1 |  |  |  |  |  |  |
| **L-Lys** | **3** | 176.8 | 57.1 | 34.1 | 25.6 | 30.7 | 42.5 |  |  |  |  |  |
| **D-Ala** | **4/5** |  | 54.2 | 20.7 |  |  |  |  |  |  |  |  |
|  |  |  |  |  |  |  |  |  |  |  |  |  |
| **PPi** |  | **P** |  |  |  |  |  |  |  |  |  |  |
| **Pi** | **1** | -5.2 |  |  |  |  |  |  |  |  |  |  |
| **Pi** | **2** | -7.7 |  |  |  |  |  |  |  |  |  |  |

**Supplementary Table T2:**

^13^C,^15^N-labelled teixobactin in the Lipid II bound state, obtained from ssNMR measurements in DOPC liposomes. Assignments in ppm. Overlapping ^13^C-signals are shown in the same color.

| **Res** | **#** | **N** | **H(N)** | **CO** | **Cα** | **Cβ** | **Cγ/Cγ1** | **Cγ2** | **Cδ** | **Cε** | **Cζ** | **MeN** | **Nε/Hε1** | **Nγ/Hγ** | **Nδ/Hδ** | **Nζ/Hζ** |
| --- | --- | --- | --- | --- | --- | --- | --- | --- | --- | --- | --- | --- | --- | --- | --- | --- |
| **N-Me-D-Phe** | **1** | 40.7 | 11.7 8.8 | 170.5 | 64.7 | 38.6 | 139.1 |  | ~132 | ~132 | ~132 | 34.1 |  |  |  |  |
| **Ile** | **2** | 128.9 | 8.9 | 174.1 | 59.6 | 43.3 | 26.4 | 18.4 | 15.4 |  |  |  |  |  |  |  |
| **Ser** | **3** | 119.7 | 9.6 | 173.2 | 57.6 | 67.1 |  |  |  |  |  |  |  |  |  |  |
| **D-Gln** | **4** | 121.0 | 9.0 | 175.1 | 55.7 | 32.8 | 34.1 |  | 181.3 |  |  |  | 111.0/  8.0/  7.3 |  |  |  |
| **D-allo-Ile** | **5** | 112.6 | 8.5 | 175.8 | 58.0 | 44.8 | 30.7 | 17.5 | 15.9 |  |  |  |  |  |  |  |
| **Ile** | **6** | 120.7 | 8.6 | 177.2 | 56.8 | 38.9 | 27.7 | 19.1 | 12.1 |  |  |  |  |  |  |  |
| **Ser** | **7** | 126.2 | 11.2 | 178.2 | 61.3 | 65.4 |  |  |  |  |  |  |  |  |  |  |
| **D-Thr** | **8** | 110.8 | 8.7 | 173.3 | 58.8 | 72.6 | 18.7 |  |  |  |  |  |  |  |  |  |
| **Ala** | **9** | 127.8 | 9.0 | 179.6 | 56.4 | 19.5 |  |  |  |  |  |  |  |  |  |  |
| **End** | **10** | 110.9 | 9.6 | 175.6 | 54.9 | 40.5 | 55.0 |  | 51.7 |  | 163.0 |  |  | 89.7/ 9.2 | 76.5/  7.5 | 69.3/ 8.2 |
| **Ile** | **11** | 117.7 | 8.7 | 173.0 | 61.3 | 39.4 | 28.2 | 18.1 | 15.6 |  |  |  |  |  |  |  |

List of overlapping ^13^C-resonances (in ppm)

1. S3CO (173.2); T8CO (173.3); I11CO (173.0)
2. S7α (61.3); I11α (61.3)
3. End10α (54.9); End10γ (55.0)
4. F1MeN (34.1); Q4γ (34.1)
5. I2γ2 (18.4); T8γ (18.7); I11γ2 (18.1)
6. I2δ (15.4); I11δ (15.6)

**Supplementary Table T3:**

Comparison of ^13^C,^15^N-chemical shifts assignments of teixobactin in complex with Lipid II in DPC micelles (ref. ^1^) and in membranes.

| **Res** | **#** | **N** | **H(N)** | **CO** | **Cαβ** | **Cγ/Cγ1** | **Cγ2** | **Cδ/Cδ1** | **Cζ** | **MeN** | **Nε/Hε1/2** | **Nγ/Hγ** | **Nδ/Hδ** | **Nζ/Hζ** |
| --- | --- | --- | --- | --- | --- | --- | --- | --- | --- | --- | --- | --- | --- | --- |
| **N-Me-D-Phe** | **1** | **-69.9** | **4.2 2.0.** | **-6.7** | 0.3 | NA | NA | NA | NA | NA |  |  |  |  |
| **Ile** | **2** | -0.6 | 0.3 | 0.9 | -0.2 | -1.1 | 0.8 | 0.4 |  |  |  |  |  |  |
| **Ser** | **3** | -0.5 | 0.3 | 0.0 | 0.0 |  |  |  |  |  |  |  |  |  |
| **D-Gln** | **4** | -0.5 | 0.2 | 0.3 | 0.2 | 0.0 |  | NA |  |  | **70.0 -3.4 -1.2** |  |  |  |
| **D-allo-Ile** | **5** | -0.6 | 0.2 | 0.1 | 0.2 | 0.4 | 0.6 | 0.6 |  |  |  |  |  |  |
| **Ile** | **6** | -0.4 | 0.2 | 0.4 | -0.2 | 0.2 | 0.3 | -0.8 |  |  |  |  |  |  |
| **Ser** | **7** | -0.5 | 0.3 | 0.6 | 0.0 |  |  |  |  |  |  |  |  |  |
| **D-Thr** | **8** | 0.1 | 0.3 | -0.1 | 0.9 |  | 0.0 |  |  |  |  |  |  |  |
| **Ala** | **9** | -0.3 | 0.2 | 0.8 | 0.2 |  |  |  |  |  |  |  |  |  |
| **End** | **10** | 0.0 | 0.4 | 0.5 | 0.3 | 0.3 |  | -0.1 | **-12.8** |  |  | 0.5  0.5 | 0.1 0.4 | -0.9 0.3 |
| **Ile** | **11** | -0.2 | 0.2 | -0.5 | -0.3 | -0.1 | 0.2 | **2.3** |  |  |  |  |  |  |

*Orange background = difference due to erroneous assignments in ref (^1^)*

*Red background = major chemical shift difference in membranes*

*NA, light blue background = not assigned in micelles*

*Cαβ = calculated as (Cα_membrane_ – Cβ_membrane_) – (Cα_micelle_ – Cβ_micelle_)*

Chemical shift (CS) differences were calculated as *CS_membrane_ - CS_micelle_*. For ^1^H and ^15^N, CS differences are small. The major CS differences for F1 and the sidechain of Q4 relate to mistaken (swapped) assignments in ref (^1^). In ref (^1^), the backbone NH_2_ signal of the F1 N-terminus is assigned at 110.6 ^15^N ppm / 7.5 & 6.8 ^1^H ppm. In reality, this signal is the NεHε_(1+2)_ signal of the Q4 sidechain. In Extended Data Fig. 1a-c, we clearly delineate the correct assignment of the F1 spin-system and show that the ^15^N-signal at 40 ppm relates to the F1 N-terminus and not the ^15^Nε-Q4 sidechain. Note that we had acquired a 2D CαN spectrum at low-temperature to clearly assign the F1 ^15^N-signal and to be certain that signals were mixed up in ref (^1^). Also note that our assignments of 111.0 ^15^N ppm for the Q4-^15^Nε signal (assigned at 41.1 ppm in micelles) matches well the to the average shift of Q-^15^Nε signal (111.9 ppm in the BMRB database (https://bmrb.io/ref_info/).

For ^13^C assignments, we observed sizeable (> 1 ppm) CS differences for the sidechains of I2 and I11 compared to micellar assignments. Intriguingly, we observed huge CS differences for the CO of F1 (-6.7 ppm) and the Cζ of End10 (-12.8 ppm), i.e., for the two residues that are prominently involved in Lipid II coordination, suggesting an impact of the membrane environment. Note that, presumably due to differences in chemical shift referencing, we subtracted 0.5 ppm for all ^13^C assignments in micelles and added to 0.2 ppm for all ^1^H assignments in micelles to improve the match to data in membranes.

**Supplementary Table T4:**

ssNMR contacts that demonstrate that teixobactin forms antiparallel β-sheets. Ambiguous contacts are coloured in green. Note that most contacts are symmetric, and they are ordered according to the amino-acid sequence.

|  | **Res** | **^13^C/ppm** | **Res** | **^13^C/ppm** |
| --- | --- | --- | --- | --- |
| 1 | F1α | 64.7 | A9α | 56.4 |
| 2 | F1aro | ~132 | A9α | 56.4 |
| 3 | F1aro | ~132 | S7α/I11α | 61.3 |
| 4 | F1aro | ~132 | S7β | 65.4 |
| 5 | I2α | 59.6 | S7α/I11α | 61.3 |
| 6 | I2β | 43.3 | S7α/I11α | 61.3 |
| 7 | I2β | 43.3 | I6α | 56.8 |
| 8 | S3β | 67.1 | S7α/I11α | 61.3 |

**
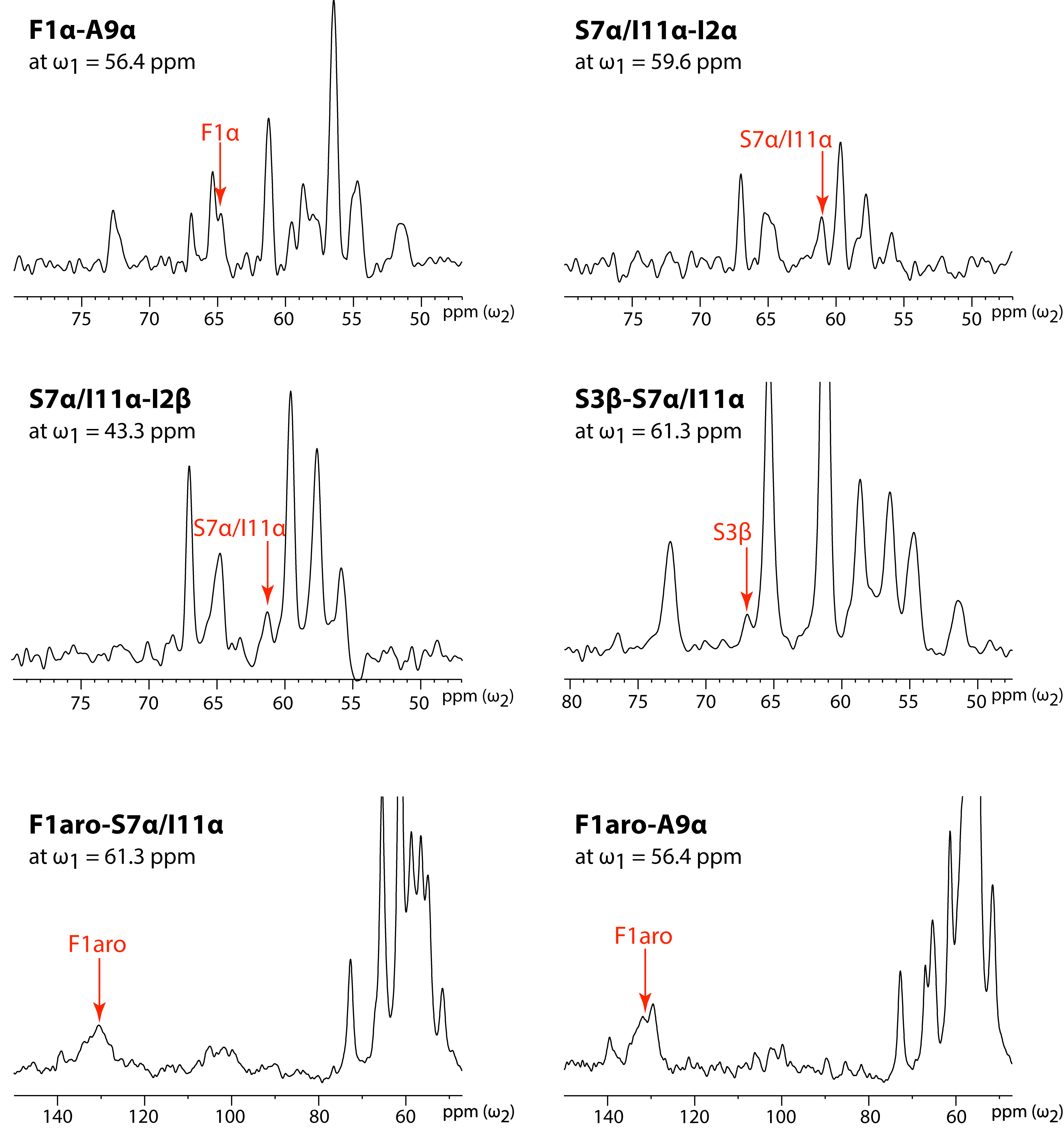
**

**Supplementary Fig. 2:**

**Horizontal cross-sections (rows) of long-range contacts from 2D CC spectra.**

Cross-sections (rows) of 2D CC spectra for selected long-range contacts corresponding to contacts 1, 2, 3, 5, 6, and 8 in Supplementary Table 4.

**
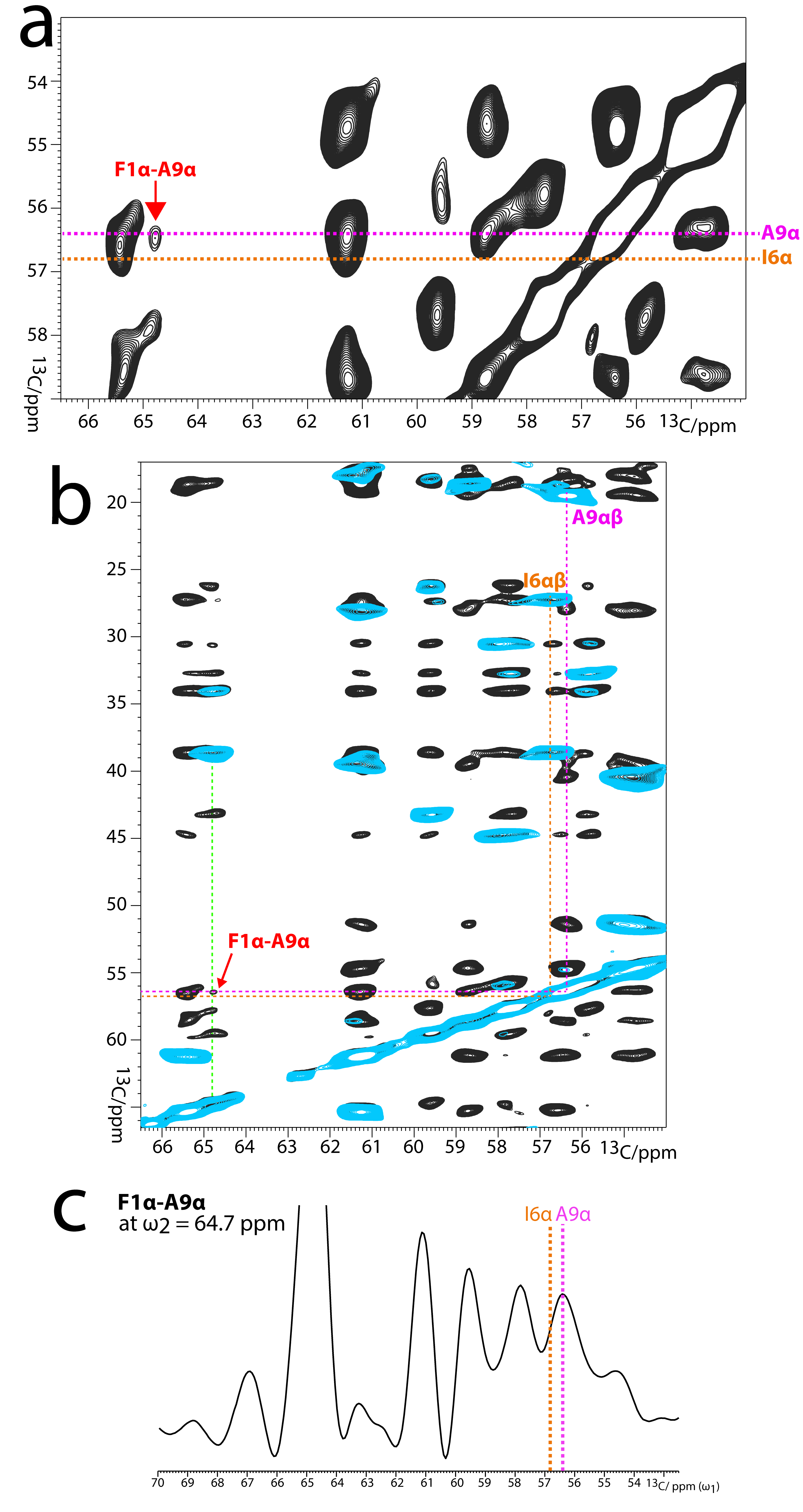
**

**Supplementary Fig. 3:**

**Unambiguous contact F1α-A9α**

A9α and I6α are separated by 0.4 ppm. Due to the high magnetic field (950 MHz) and the high-spectral quality, we could unambiguously assign cross-peaks involving A9α or I6α, as shown by the example of the cross-peak F1α-A9α. a,b) Spectral cut-outs of 2D PARISxy^2^ CC spectra acquired with 50 (cyan) and 600 (black) ms CC mixing time. c) Vertical cross-section (column) at 64.7 ^13^C ppm, i.e., the position of the F1α signal.

**SUPPLEMENTARY DISSCUSION**

**NMR restraints and Structure validation**

Dihedral angle restraints (intramolecular): Dihedral restraints were obtained using the TALOS-N^3^ software. Only restraints with ‘Good’ accuracy (i.e., the best accuracy in TALOS-N), were considered. Dihedral angle restraints were applied for Ile2-Ser6 of teixobactin. Restraints were implemented with boundaries of +/- 20°.

Intramolecular Lipid II distance restraints: We only considered potentially meaningful distance restraints between the sugars or between sugars and the pentapeptide. Contacts were defined with upper and lower limits of 6.0 and 1.5 Å, respectively, based on a series of 2D ^13^C^13^C PARISxy experiments.

Intermolecular distance restraints between teixobactin molecules

All restraints based on a series of 2D ^13^C^13^C PARISxy experiments (up to 600 ms ^13^C^13^C transfer) using a target distance of 7.5 Å, and lower distance margin of 5.5 Å, and a higher distance margin of 1.5 Å; i.e., format (7.5 5.5 1.5). For a few weak signals, we used the format (8.5 6.5 1.5).

Note that in the β-sheet of four teixobactin molecules, intermolecular teixobactin-teixobactin distance restraints for the *inner* teixobactin molecules were implemented so that a restraint could be with the teixobactin molecule on either side.

Detailed list of teixobactin – teixobactin restraints.

**Supplementary Table T5:**

**Unambiguous**

| **#** |  | **^13^C-CS (ppm)** |  | **^13^C-CS (ppm)** | **Average shortest intramolecular distance (Å)** | **Average shortest intermolecular distance (Å)** |
| --- | --- | --- | --- | --- | --- | --- |
| 1 | Phe1Cα | 64.7 | Ile5Cβ | 44.8 | 12.3 ± 0.7 | 7.5 ± 0.4 |
| 2 | Phe1Cα | 64.7 | Ile5Cγ1 | 30.7 | 12.5 ± 1.0 | 7.8 ± 0.4 |
| 3 | Phe1Cγ | 139.1 | Ile5Cγ1 | 30.7 | 13.8 ± 1.8 | 9.3 ± 1.2 |
| 4 | Phe1CO | 170.5 | Ile5Cβ | 44.8 | 11.2 ± 0.6 | 6.9 ± 0.3 |
| 5 | Phe1CO | 170.5 | Ile5Cγ1 | 30.7 | 11.4 ± 0.9 | 7.4 ± 0.3 |
| 6 | Phe1Cα | 64.7 | Ile6Cα | 56.8 | 16.2 ± 0.7 | 5.5 ± 0.4 |
| 7 | Phe1Cα | 64.7 | Ile6Cγ1 | 27.7 | 17.3 ± 1.4 | 6.2 ± 1.2 |
| 8 | Phe1Cα | 64.7 | Ile6Cδ | 12.1 | 17.6 ± 1.5. | 6.6 ± 1.6 |
| 9 | Phe1Cγ | 139.1 | Ile6Cγ1 | 27.7 | 18.4 ± 2.1 | 7.4 ± 1.6 |
| 10 | Phe1Cγ | 139.1 | Ile6Cδ | 12.1 | 18.6 ± 2.2 | 7.6 ± 2.1 |
| 11 | Phe1CO | 170.5 | Ile6Cγ1 | 27.7 | 16.1 ± 1.2 | 6.4 ± 1.0 |
| 12 | Phe1CO | 170.5 | Ile6Cδ | 12.1 | 16.4 ± 1.3 | 6.4 ± 1.2 |
| 13 | Phe1Cα | 64.7 | Ala9Cα | 56.4 | 24.8 ± 1.1 | 4.9 ± 0.8 |
| 14 | Ile2Cα | 59.6 | Ile6Cγ1 | 27.7 | 13.9 ± 1.2 | 5.1 ± 0.7 |
| 15 | Ile2Cα | 59.6 | Ile6Cδ | 12.1 | 14.3 ± 1.2 | 4.9 ± 0.9 |
| 16 | Ile2Cα | 59.6 | Ile6CO | 177.2 | 14.0 ± 0.5 | 5.0 ± 0.2 |
| 17 | Ile2Cβ | 43.3 | Ile6Cδ | 12.1 | 13.5 ± 1.5 | 4.1 ± 0.5 |
| 18 | Ile2Cβ | 43.3 | Ile6Cγ1 | 27.7 | 13.2 ± 1.4 | 4.5 ± 0.5 |
| 19 | Ile2Cγ1 | 26.4 | Ile6CO | 177.2 | 13.4 ± 1.2 | 5.2 ± 1.0 |
| 20 | Ile2Cγ1 | 26.4 | Ile6Cδ | 12.1 | 13.6 ± 2.0 | 4.6 ± 0.6 |
| 21 | Ile2Cβ | 43.3 | Ile6Cα | 56.8 | 12.2 ± 0.9 | 5.6 ± 0.4 |

The shortest distances were averaged over the 25 structures of the ensemble. The error shows the standard deviation. Without exception, intramolecular distances are substantially longer than intramolecular distances.

In addition, we applied 11 ambiguous intermolecular teixobactin - teixobactin distance restraints:

**Supplementary Table T6:**

| **Ambiguous** |  |  |  |  |  |  |
| --- | --- | --- | --- | --- | --- | --- |
| **#** |  | **^13^C-CS**  **(ppm)** |  | **^13^C-CS**  **(ppm)** | **Average shortest intramolecular distance (Å)** | **Average shortest intermolecular distance (Å)** |
| 1 | Phe1Caro | ~132 | Ser7Cβ | 65.4 | 21.5 ± 1.9 | 6.4 ± 1.2 |
| 2 | Phe1Caro | ~132 | Ser7Cα | 61.3 | 21.3 ± 2.0 | 7.1 ± 0.8 |
|  | Phe1Caro | ~132 | Ile11Cα | 61.3 | 28.0 ± 1.7 | 7.8 ± 2.6 |
| 3 | Phe1Caro | ~132 | Ala9Cα | 56.4 | 26.2 ± 2.4 | 5.7 ± 1.4 |
| 4 | Phe1Cδ,ε,ζ | ~132 | Ile6Cγ1 | 27.7 | 18.8 ± 2.8 | 8.2 ± 2.0 |
| 5 | Phe1Cδ,ε,ζ | ~132 | Ile6Cδ1 | 12.1 | 18.9 ± 2.9 | 8.0 ± 2.5 |
| 6 | Phe1Cδ,ε,ζ | ~132 | Ile5Cγ1 | 30.7 | 14.2 ± 2.4 | 10.0 ± 1.8 |
| 7 | Phe1Cδ,ε,ζ | ~132 | Ile5Cβ | 44.8 | 14.1 ± 2.1 | 9.8 ± 1.6 |
| 8 | Ile2Cα | 59.6 | Ser7Cα | 61.3 | 13.2 ± 0.3 | 5.0 ± 0.5 |
|  | Ile2Cα | 59.6 | Ile11Cα | 61.3 | 19.9 ± 0.7 | 9.5 ± 0.7 |
| 9 | Ile2Cβ | 43.3 | Ser7Cα | 61.3 | 13.8 ± 0.2 | 5.3 ± 0.4 |
|  | Ile2Cβ | 43.3 | Ile11Cα | 61.3 | 20.3 ± 0.9 | 9.9 ± 0.6 |
| 10 | Ser3Cβ | 67.1 | Ser7Cα | 61.3 | 14.4 ± 0.4 | 5.6 ± 0.6 |
|  | Ser3Cβ | 67.1 | Ile11Cα | 61.3 | 20.7 ± 1.1 | 9.5 ± 0.7 |
| 11 | Phe1MeN | 34.1 | Ser7Cα | 61.3 | 20.5 ± 0.8 | 3.7 ± 0.5 |
|  | Phe1MeN | 34.1 | Ile11Cα | 61.3 | 26.9 ± 1.0 | 7.5 ± 1.2 |
|  | Gln4Cγ | 34.1 | Ser7Cα | 61.3 | 10.3 ± 0.7 | 7.5 ± 0.8 |
|  | Gln4Cγ | 34.1 | Ile11Cα | 61.3 | 16.3 ± 0.8 | 12.5 ± 0.9 |

The shortest distances were averaged over the 25 structures of the ensemble. The error shows the standard deviation. Without exception, intramolecular distances are substantially longer than intramolecular distances.

Note that for the aromatic ring (Phe1Caro / Phe1Cδ,ε,ζ), we measured for simplicity the shortest distances involving the centre of mass of the aromatic ring, while the actual distances are shorter. I.e., for contact Phe1Cδ,ε,ζ - Ile5Cγ1, the shortest distance involving the centre of mass is 10.0 ± 1.8 Å, while the precise distance is shorter (9.1 ± 1.5 Å). Likewise, for restraint Phe1Cδ,ε,ζ - Ile5Cβ, the distance involving the centre of mass is 9.8 ± 1.6 Å, while the precise distance is 8.8 ± 1.3 Å.

Note that we applied contacts between residues Ile5 or Ile6 and the aromatic ring as unambiguous contacts with Phe1Cγ and ambiguous contacts with the rest of the aromatic ring. For residues Ser7/Ile11, we defined only ambiguous contacts with the entire aromatic ring, which provided best results.

Hydrogen bond restraints (intermolecular) between teixobactin molecules: Hydrogen bonding restraints in line with experimentally determined antiparallel *teixobactin* β-sheets were applied. Antiparallel β-sheets were conclusively shown for R4L10-teixobactin. For natural teixobactin, ssNMR data (Fig. 1c,d of the manuscript and Extended Data Fig. 3 and 4) also fully consistent with antiparallel β-sheets, which is further corroborated by fluorescence spectroscopy data (Extended Data Fig. 2b,c). While three different register shifts are possible to form antiparallel teixobactin β-sheets (i.e., Ser3NH could interact with Ser7CO, Ile5CO, or Ile3CO), only the variant with Ser7CO agrees with the intermolecular ssNMR distance restraints. Hydrogen bonding distance restraints were defined accordingly with upper and lower limits of 2.3 and 1.5 Å, respectively, i.e., format (2.0 0.5 0.3).

*teixobactin* ***A*** *with teixobactin* ***B***

Ser3NH - Ser7CO

Ser3CO - Ser7NH

Ile5NH - Ile5CO

Ile5CO - Ile5NH

Ser7NH - Ser3CO

Ser7CO - Ser3NH

*teixobactin* ***B*** *with teixobactin* ***C***

Ile2NH - Ile6CO

Ile2CO - Ile6NH

Arg4NH - Arg4CO

Arg4CO - Arg4NH

Ile6NH - Ile2CO

Ile6CO - Ile2NH

*teixobactin* ***C*** *with teixobactin* ***D***

Similar as in ***A*** with ***B***

Intermolecular distance restraints between teixobactin and Lipid II: *Restraints involving the pyrophosphate group:* Ambiguous distance restraints were applied between the backbone amino protons of the four depsi-cycle residues (Thr8-Ile11) with either phosphate of the pyrophosphate group using upper and lower limits of 2.4 and 1.7 Å, respectively, i.e., format (2.0 0.3 0.4). Restraints based on a 2D ^1^H^31^P experiment (Fig. 3b of the manuscript).

*Restraints involving the MurNAc/GlcNAc sugars and A1 of the pentapeptide:* Restraints were based on a series of 2D ^13^C^13^C PARISxy experiments with 150 and 300 ms magnetization transfer. Interfacial restraints were applied using three different formats. For interfacial signals that we could clearly assign with the shorter transfer time of 150 ms, we used tighter boundaries, i.e., format (5.0 4.0 1.0), implying upper and lower limits of 6.0 and 1.0 Å, respectively. For all other interfacial restraints, we used the format (6.0 5.0 1.5), with the exception of weak contacts, for which wider boundaries were applied (7.5 6.0 1.5), i.e., upper and lower boundaries of 9.0 and 1.5 Å, respectively.

****: We obtained 13 contacts that were unambiguous between a certain residue of teixobactin and MurNAc. Some of these contacts, highlighted in grey shading below, involved i) the Cα and Cγ of End10 or ii) C3 and C9 of MurNAc, which have similar chemical shifts.

**Supplementary Table T7:**

Detailed list of teixobactin – Lipid II restraints.

| **#** |  | **^13^C-CS (ppm)** |  | **^13^C-CS (ppm)** | **Average shortest interfacial distance (Å)** |
| --- | --- | --- | --- | --- | --- |
| 1 | Mu6C1 | 98.9 | Ala9Cβ | 19.5 | 4.2 ± 0.6 |
| 2 | Mu6C1 | 98.9 | End10Cα | 54.9 | 5.5 ± 0.7 |
|  | Mu6C1 | 98.9 | End10Cγ | 55.0 | 4.8 ± 0.7 |
| 3 | Mu6C1 | 98.9 | End10Cβ | 40.5 | 5.3 ± 0.4 |
| 4 | Mu6C1 | 98.9 | End10Cζ | 163.0 | 4.7 ± 0.3 |
| 5 | Mu6C1 | 98.9 | Phe1MeN | 34.1 | 6.1 ± 0.5 |
|  | Mu6C1 | 98.9 | Gln4Cγ | 34.1 | 14.4 ± 1.2 |
| 6 | Mu6C3 | 81.7 | Ala9Cβ | 19.5 | 6.1 ± 0.6 |
|  | Mu6C9 | 81.3 | Ala9Cβ | 19.5 | 6.8 ± 1.2 |
| 7 | Mu6C3 | 81.7 | End10Cζ | 163.0 | 4.7 ± 0.9 |
|  | Mu6C9 | 81.3 | End10Cζ | 163.0 | 4.4 ± 0.7 |
| 8 | Mu6C3 | 81.7 | Phe1MeN | 34.1 | 7.4 ± 0.7 |
|  | Mu6C9 | 81.3 | Phe1MeN | 34.1 | 8.7 ± 1.4 |
|  | Mu6C3 | 81.7 | Gln4Cγ | 34.1 | 15.9 ± 1.4 |
|  | Mu6C9 | 81.3 | Gln4Cγ | 34.1 | 16.9 ± 1.6 |
| 9 | Mu6C3 | 81.7 | End10Cα | 54.9 | 6.8 ± 1.0 |
|  | Mu6C9 | 81.3 | End10Cα | 54.9 | 7.1 ± 1.2 |
|  | Mu6C3 | 81.7 | End10Cγ | 55.0 | 6.0 ± 1.0 |
|  | Mu6C9 | 81.3 | End10Cγ | 55.0 | 5.9 ± 1.1 |
| 10 | Mu6C4 | 74.4 | End10Cζ | 163.0 | 5.1 ± 0.5 |
| 11 | Mu6C5 | 77.6 | Phe1MeN | 34.1 | 7.3 ± 0.9 |
|  | Mu6C5 | 77.6 | Gln4Cγ | 34.1 | 15.2 ± 1.9 |
| 12 | Mu6C5 | 77.6 | End10Cα | 54.9 | 6.6 ± 1.0 |
|  | Mu6C5 | 77.6 | End10Cγ | 55.0 | 6.1 ± 0.8 |
| 13 | Mu6C5 | 77.6 | End10Cδ | 51.7 | 6.0 ± 0.8 |
| 14 | Mu6C5 | 77.6 | End10Cζ | 163.0 | 4.9 ± 0.8 |
| 15 | Mu6C6 | 62.5 | End10Cζ | 163.0 | 4.2 ± 0.9 |
| 16 | Ala1*Cα | 52.7 | End10Cζ | 163.0 | 4.7 ± 0.8 |
| 17 | Mu6C8 | 25.9 | End10Cζ | 163.0 | 5.2 ± 1.4 |
|  | Gl7C8 | 26.0 | End10Cζ | 163.0 | 7.6 ± 1.4 |

The shortest distances were averaged over the 25 structures of the ensemble. The error shows the standard deviation.

Topological restraints: Eventually, a filtering strategy was applied to constrain the conformational space of the isoprenyl tails. Structures were only accepted if all Lipid II tails pointed into the direction of the membrane-anchoring residues Ile2, Ile5, and Ile6 (see Fig. 3f of the main text and Extended Data Fig. 8). Sorting of the Lipid II tails was steered by imposing soft distance restraints between the sidechain of Ile6 and the Lipid II isoprenyl-tail, and by imposing soft distance restraints between the isoprenyl-tails.

**Supplementary Table T8:**

Number and type of restraints used for the structure calculations. Intramolecular restraints are listed per monomers, while intermolecular/interfacial restraints are listed per pair of interacting molecules.

| ***Number of Restraints*** | | |
| --- | --- | --- |
| ***Intramolecular*** | *Unambiguous* | *Ambiguous* |
| Teixobactin dihedral restraints* | 10 | 0 |
| Lipid II distance restraints | 7 | 4 |
|  | | |
| ***Intermolecular***  ***Teixobactin - teixobactin*** | *Unambiguous* | *Ambiguous* |
| Distance restraints** | 21 | 11 |
| Hydrogen bonds*** | 6 | 0 |
|  | | |
| ***Intermolecular***  ***Teixobactin - Lipid II*** | *Unambiguous* | *Ambiguous* |
| Distance restraints with sugars | 13***** | 4 |
| Distance restraints with PPI**** | 0 | 4 |

*,**,***,****,***** See Methods (NMR Structure calculations) for details

**Analysis of calculated structures** (see also Supplementary Table 10)

Structural and violation statistics of the final 25 structures are given below:

Structure ensemble precision:

- Average backbone RMSD (from the average structure) of the 25 teixobactin molecules in the complex: 2.3 +/- 0.6 Å.

Violation analysis:

- *Intramolecular Lipid II distance restraints:* No violations.
- *Dihedral teixobactin restraints:* 6 of 10 chemical shift-derived dihedral restraints were fulfilled in >90 % of the calculated structures in the complex. The phi restraints for Ile5 and Ile6 were violated by 10 - 20 degrees in most calculated structures. The Gln4 psi restraint was fulfilled in 42 % of the structures and violated by about 9 degrees in the remaining structures. The Ile5 psi restraint was fulfilled in 65 % of the structures and violated by about 7 degrees in the remaining structures.
- *Intermolecular teixobactin* – *teixobactin restraints:*
- Distance restrains were generally very well fulfilled. No violations for >90 % of restraints.
- Hydrogen bond restraints were generally very well fulfilled in the ensemble. No violations for >99 % of the restraints.
- Intermolecular *teixobactin* *– Lipid II restraints*
- Distance restraints between the amino-protons of the depsi-cycle and the PPi group of Lipid II were generally very well fulfilled in the ensemble. No violations for >99 % of the restraints.
- Other interfacial distance restraints were generally very well fulfilled. Most violations were observed for the contacts between F1MeN/Q4γ and GlcNAc, between Ala9Cβ - MurNAcC3 (tight restraint with format 5.0 4.0 1.0), and End10Cζ – MurNAcC6 (tight restraint with format 5.0 4.0 1.0). However, these restrains were still fulfilled for >84 % of the calculated structures in the complex.

**Details of 2D and 3D ssNMR experiments**

1. 2D CC PARISxy experiment with ^13^C,^15^N-teixobactin – ^12^C,^14^N-Lipid II

Magnetic field / MAS = 950 MHz (^1^H-frequency) / 18 kHz

Mixing time (CC) = 50 ms

t1 points / AQ = 290 / 4.46 ms

Recycle delay = 1.81 s

Co-added transients = 304

Experimental time = 1d 22h

2. 2D CC PARISxy experiment with ^13^C,^15^N-teixobactin – ^12^C,^14^N-Lipid II

Magnetic field / MAS = 950 MHz (^1^H-frequency) / 18 kHz

Mixing time (CC) = 600 ms

t1 points / AQ = 250 / 3.84 ms

Recycle delay = 1.81 s

Co-added transients = 1072

Experimental time = 7d 13h

3. 2D CC PARISxy experiment with ^13^C,^15^N-teixobactin – ^13^C,^15^N-Lipid II

Magnetic field / MAS = 950 MHz (^1^H-frequency) / 18 kHz

Mixing time (CC) = 150 ms

t1 points / AQ = 290 / 4.46 ms

Recycle delay = 1.81 s

Co-added transients = 1232

Experimental time = 8d 6h

4. 2D CC PARISxy experiment with ^13^C,^15^N-teixobactin – ^13^C,^15^N-Lipid II

Magnetic field / MAS = 950 MHz (^1^H-frequency) / 18 kHz

Mixing time (CC) = 300 ms

t1 points / AQ = 290 / 4.46 ms

Recycle delay = 1.81 s

Co-added transients = 1232

Experimental time = 8d 19h

5. 2D T2-edited H(H)C experiment with ^13^C,^15^N-teixobactin – ^12^C,^14^N-Lipid II

Magnetic field / MAS = 700 MHz (^1^H-frequency) / 16.5 kHz

Mixing time (HH) = 5 ms

T2 filter = 2.5 ms

t1 points / AQ = 50 / 2.97 ms

Recycle delay = 1.60 s

Co-added transients = 8192

Experimental time = 7d 17h

6. 2D CC TOBSY experiment with ^13^C,^15^N-teixobactin – ^13^C,^15^N-Lipid II

Magnetic field / MAS = 700 MHz (^1^H-frequency) / 8 kHz

Mixing time (CC) = 6 ms

t1 points / AQ = 148 / 3.00 ms

Recycle delay = 1.61 s

Co-added transients = 2560

Experimental time = 7d 6h

7. 2D NC experiment with ^13^C,^15^N-teixobactin – ^12^C,^14^N-Lipid II

Magnetic field / MAS = 700 MHz (^1^H-frequency) / 12 kHz

Mixing time (NC) = 5 ms

t1 points / AQ = 78 / 4.99 ms

Recycle delay = 2.20 s

Co-added transients = 1536

Experimental time = 3d 2h

8. 2D NH experiment with ^13^C,^15^N-teixobactin – ^12^C,^14^N-Lipid II

Magnetic field / MAS = 700 MHz (^1^H-frequency) / 60 kHz

t1 points / AQ = 144 / 9.80 ms

Recycle delay = 0.80 s

Co-added transients = 1888

Experimental time = 3d 2h

Note = high number of co-added transients was necessary to obtain high quality data for sidechains of residues 1 and 4.

9. 3D CONH experiment with ^13^C,^15^N-teixobactin – ^12^C,^14^N-Lipid II

Magnetic field / MAS = 700 MHz (^1^H-frequency) / 60 kHz

t1 points / AQ = 26 / 2.95 ms (25 ^13^C ppm)**

t2 points / AQ = 48 / 6.50 ms (52 ^15^N ppm*)**

Recycle delay = 0.81 s

Co-added transients = 1088

Experimental time = 5d 10h

**large ^15^N spectral width to detect the sidechain nitrogens of End10*

***using 35% non-uniform sampling in indirect dimensions*

10. 3D CaNH experiment with ^13^C,^15^N-teixobactin – ^12^C,^14^N-Lipid II

Magnetic field / MAS = 700 MHz (^1^H-frequency) / 60 kHz

t1 points / AQ = 24 / 3.40 ms (SW = 20 ^13^C ppm)**

t2 points / AQ = 30 / 7.29 ms (SW = 29 ^15^N ppm)**

Recycle delay = 0.81 s

Co-added transients = 1120

Experimental time = 4d 1h

***using 35% non-uniform sampling in indirect dimensions*

*For all 2D and 3D experiments, sign discrimination in indirect dimensions was achieved with the TPPI (time-proportional phase incrementation) method.*

**Calculation of partition coefficient logP**

The input structure for the logP calculation was built by connecting the three hydrophobic sidechains of residues Ile2, Ile5, and Ile6 to a single carbon centre, as shown in the Figure below. The logP calculations were performed with two different software packages ChemDraw Ultra 12.0 and Maestro 12.7 (Schrödinger Release 2021-1: Maestro Version 12.7.156, Schrödinger, LLC, New York, NY, 2021), which gave similar logP values of 5.57 (ChemDraw) and 5.48 (Maestro).

Both software packages calculate logP using the equation below, as described in ref (^4^), which is ref. 38 of the manuscript.

$\log P= \sum_{i} n_{i}a_{i}$

where n_i_ is the number of atoms of type i and a_i_ is the atomic logP contribution.


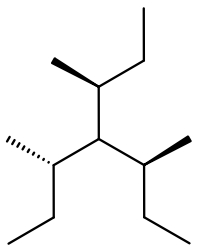


Input structure used for the logP calculation.

**Supplementary Table T9:**

Difference of the i) chemical shifts of natural teixobactin in complex with Lipid II and ii) the previously^5^ published chemical shifts of R4L10-teixobactin in complex with Lipid II. Both systems were acquired in DOPC liposomes with 4 % Lipid II. All values in ppm.

| **Res** | **#** | **N** | **H(N)** | **CO** | **Cα** | **Cβ** | **Cγ/Cγ1** | **Cγ2** | **Cδ** |
| --- | --- | --- | --- | --- | --- | --- | --- | --- | --- |
| **Ile** | **2** | 0,6 | 0,1 | -0,9 | 0,0 | -0,5 | 1,3 | -1,2 | 0,0 |
| **Ser** | **3** | 0,0 | -0,3 | -0,3 | -0,1 | 0,0 |  |  |  |
| **Ile** | **6** | 0,5 | -0,2 | -0,6 | 0,9 | 0,0 | -0,5 | -0,6 | 0,6 |
| **Ser** | **7** | 0,9 | 0,4 | -0,8 | 0,1 | 0,1 |  |  |  |
| **Ala** | **9** | -1,0 | -0,4 | -1,2 | -0,2 | -0,5 |  |  |  |

Note that, due to small differences in chemical shift referencing, we added 0.4 ppm for all ^13^C assignments of R4L10-teixobactin to improve the match to the chemical shifts of natural teixobactin. Chemical shift (CS) differences were calculated as *CS_R4L10-teixobactin_ – CS_teixobactin_*.

**Supplementary Table T10:**

**NMR and refinement statistics for protein structures**

|  | **Protein** |
| --- | --- |
| **NMR distance and dihedral constraints** |  |
| Distance constraints |  |
| Total NOE | 64 |
| Intra-residue | 0 |
| Inter-residue | 11 |
| Sequential (\|*i* – *j*\| = 1) | 11 |
| Medium-range (\|*i* – *j*\| < 4) | 0 |
| Long-range (\|*i* – *j*\| > 5) | 0 |
| Intermolecular | 53 |
| Hydrogen bonds | 6 |
| Total dihedral angle restraints | 10 |
| φ | 5 |
| ψ | 5 |
|  |  |
| **Structure statistics** |  |
| Violations (mean and s.d.) |  |
| Distance restraints (Å) | 0.16 +/- 0.08 |
| Dihedral angle restraints (º) | 8.18 +/- 1.28 |
| Max. dihedral angle violation (º) | 23.76 |
| Max. distance constraint violation (Å) | 2.82 |
| Deviations from idealized geometry |  |
| Bond lengths (Å) | 0.042 +/- 0.003 |
| Bond angles (º) | 5.75 +/- 0.19 |
| Impropers (º) | 7.55 +/- 0.98 |
| Average pairwise r.m.s. deviation** (Å) |  |
| Heavy | 2.8 +/- 0.5 Å |
| Backbone | 2.3 +/- 0.6 Å |

**Pairwise r.m.s. deviation was calculated among 25 refined structures.

**Supplementary Video 1**

Assembly of teixobactin – Lipid II fibrils as captured by HS-AFM on a DOPC-lipid bilayer containing 1% Lipid II in presence of 800 nM teixobactin. Imaging speed 0.5 frame/s.

**Supplementary Video 2**

Assembly of teixobactin – Lipid II fibrils as captured by HS-AFM on a DOPC-lipid bilayer containing 1% Lipid II in presence of 1µM teixobactin. Imaging speed 2 frames/s.

**Supplementary Video 3**

Animation of 3D rendered images of membrane deformed by teixobactin – Lipid II fibrils shown from different angles. The z scale bar (1 nm) is relative and only refers to the depicted top part of the membrane. The 3D image frame dimensions are 265 nm in X-Y and 0.5 nm in Z.

**References**

1 Oster, C. *et al.* Structural studies suggest aggregation as one of the modes of action for teixobactin. *Chem Sci* **9**, 8850-8859, doi:10.1039/c8sc03655a (2018).

2 Weingarth, M., Bodenhausen, G. & Tekely, P. Broadband magnetization transfer using moderate radio-frequency fields for NMR with very high static fields and spinning speeds. *Chemical Physics Letters* **488**, 10-16, doi:10.1016/j.cplett.2010.01.072 (2010).

3 Shen, Y. & Bax, A. Protein backbone and sidechain torsion angles predicted from NMR chemical shifts using artificial neural networks. *Journal of Biomolecular NMR* **56**, 227-241, doi:10.1007/s10858-013-9741-y (2013).

4 Ghose, A. K., Viswanadhan, V. N. & Wendoloski, J. J. Prediction of Hydrophobic (Lipophilic) Properties of Small Organic Molecules Using Fragmental Methods:  An Analysis of ALOGP and CLOGP Methods. *The Journal of Physical Chemistry A* **102**, 3762-3772, doi:10.1021/jp980230o (1998).

5 Shukla, R. *et al.* Mode of action of teixobactins in cellular membranes. *Nat Commun* **11**, 2848, doi:10.1038/s41467-020-16600-2 (2020).
